# The impact of CBP expression in estrogen receptor-positive breast cancer

**DOI:** 10.1186/s13148-021-01060-2

**Published:** 2021-04-07

**Authors:** Wafaa S. Ramadan, Iman M. Talaat, Mahmood Y. Hachim, Annette Lischka, Timo Gemoll, Raafat El-Awady

**Affiliations:** 1 Sharjah Institute for Medical Research, University of Sharjah, Sharjah, United Arab Emirates; 2 College of Medicine, University of Sharjah, Sharjah, United Arab Emirates; 3 Department of Pathology, Faculty of Medicine, University of Alexandria, Alexandria, Egypt; 4 College of Medicine, Mohammed Bin Rashid University of Medicine and Health Sciences, Dubai, United Arab Emirates; 5 Section for Translational Surgical Oncology and Biobanking, Department of Surgery, University of Lübeck and University Hospital Schleswig-Holstein, Campus Lübeck, Lübeck, Germany; 6 College of Pharmacy, University of Sharjah, Sharjah, United Arab Emirates

**Keywords:** Breast cancer, CBP, *CREBBP*, GCN5, *KAT2A*, ERα, HER2, Luminal subtypes

## Abstract

**Background:**

The development of new biomarkers with diagnostic, prognostic and therapeutic prominence will greatly enhance the management of breast cancer (BC). Several reports suggest the involvement of the histone acetyltransferases CREB-binding protein (CBP) and general control non-depressible 5 (GCN5) in tumor formation; however, their clinical significance in BC remains poorly understood. This study aims to investigate the value of CBP and GCN5 as markers and/or targets for BC prognosis and therapy. Expression of CBP, GCN5, estrogen receptor α (ERα), progesterone receptor (PR) and human epidermal growth factor receptor 2 (HER2) in BC was analyzed in cell lines by western blot and in patients’ tissues by immunohistochemistry. The gene amplification data were also analyzed for CBP and GCN5 using the publicly available data from BC patients.

**Results:**

Elevated expression of CBP and GCN5 was detected in BC tissues from patients and cell lines more than normal ones. In particular, CBP was more expressed in luminal A and B subtypes. Using chemical and biological inhibitors for CBP, ERα and HER2 showed a strong association between CBP and the expression of ERα and HER2. Moreover, analysis of the *CREBBP* (for CBP) and *KAT2A* (for GCN5) genes in a larger number of patients in publicly available databases showed amplification of both genes in BC patients. Amplification of *CREBBP* gene was observed in luminal A, luminal B and triple-negative but not in HER2 overexpressing subtypes. Furthermore, patients with high *CREBBP* or *KAT2A* gene expression had better 5-year disease-free survival than the low gene expression group (*p* = 0.0018 and *p* < 0.00001, respectively).

**Conclusions:**

We conclude that the persistent amplification and overexpression of CBP in ERα- and PR-positive BC highlights the significance of CBP as a new diagnostic marker and therapeutic target in hormone-positive BC.

**Supplementary Information:**

The online version contains supplementary material available at 10.1186/s13148-021-01060-2.

## Background

Breast cancer (BC) is the most common type of malignancy among females accounting for approximately 2.1 million new cases and 0.6 million deaths reported in 2018 worldwide [1]. Management of BC depends largely on enhancing the outcome and survival of patients through early detection of the disease. The increased BC mortality during the past 25 years could be attributed to the high percentage of patients who are still diagnosed at advanced stages [2–4].

In addition, the cure rates of the currently available BC treatment modalities are highly dependent on the molecular subtype of the tumor and the stage at diagnosis, which, in some cases, do not result in satisfactory clinical outcomes [5]. Inherent and/or acquired resistance to the existing hormonal and non-hormonal BC therapeutics is the main reason for BC therapy failure [6, 7]. The great advances in understanding the biology and pathogenesis of BC lead to the development of targeted BC therapeutics. Such therapeutics are targeting molecules such as the human epidermal growth factor receptor 2 (HER2), the phosphoinositide-3-kinase (PI3K), the vascular endothelial growth factor (VEGF), the epidermal growth factor receptor (EGFR), the programmed death-1 (PD-1), the poly (adenosine diphosphate-ribose) polymerase (PARP), or the cyclin-dependent kinases [8]. Despite this arena of BC therapeutics, resistance and disease relapse is still an issue in some cases. Thus, the search for new biomarkers with diagnostic, prognostic and therapeutic purposes is still needed to assist in the clinical management of BC patients [9]. Currently, the BC diagnosis and treatment decisions are mainly based on the expression of hormone receptors such as ER, PR and the expression status of HER2. Epigenetic modifications in cancer cells are now recognized to play an essential role in carcinogenesis and in the response of cells to cancer therapy. The development of epigenetic markers can therefore largely improve the outcome of advanced BC [10].

Acetylation of histone and non-histone proteins is an important epigenetic factor that regulates diverse biological processes related to DNA replication, transcription, DNA repair, cell growth and death [11]. The addition of an acetyl group to lysine residues is catalyzed by histone acetyltransferases (HATs), while this addition is reversed by the function of histone deacetylases (HDACs). Modification of the acetylation profile of proteins in cancer cells through mutations, overexpression, or dysfunction of these two families of enzymes is well known to contribute to the pathological program of carcinogenesis [12]. Besides, the reversible and dynamic nature of histone acetylation provides a therapeutic window of opportunity [13]. Therefore, it is essential to study the role of these epigenetic regulators in the context of tumorigenesis to find a suitable epigenetic factor serving as a biomarker as well as a therapeutic target.

General control non-depressible 5 (GCN5) and CREB-binding protein (CBP) are HATs that are reported to play a key role in various types of cancers [12]. The overexpression of GCN5 has been reported in lung, colon, liver, endometrial cancers as well in Burkitt’s lymphoma and glioma [14–19]. Indeed, it was found to exert an oncogenic role through the acetylation of oncoproteins like c-MYC, AIB1 and the translocated E2A-PBX1 [16, 20, 21]. It also plays a fundamental role in mediating diverse malignant processes such as cell cycle perturbations, cell migration and DNA damage repair [15, 22, 23]. On the other hand, several reports mentioned the involvement of the CBP in both tumor-suppression and oncogenesis pathways, which forms a paradox about the function of CBP in cancer [12, 24, 25]. The status of CBP in cancer was found to be diverse, linked to chromosomal translocation in acute myeloid leukemia, somatic mutations in ovarian cancer and overexpression in lung and colon cancers [26–29].

Currently, very few reports are available about the status of the CBP and the GCN5 in BC. A recent study suggested a role for CBP in the biology of triple negative BC [30]. Both HATs were reported previously in regulating the estrogen receptor signaling pathway that is implicated in breast carcinogenesis [31]. In particular, CBP/p300 is a well-known coactivator that functions in stimulating the transcriptional activity of the ER to induce the expression of estrogen-response elements [32–34]. Also, CBP was previously investigated as a potential target in metastatic BC [35].

The aim of this study was to investigate the expression status of CBP and GCN5 in BC patients’ tissues and BC cell lines compared to their normal counterparts. Also, to study the CBP and GCN5 expression profiles in different subtypes of BC and their association with the different clinicopathological parameters. The ultimate goal was to investigate the possibility of using CBP and/or GCN5 as markers and targets for BC prognosis and therapy.

## Results

### CBP and GCN5 expression in breast cell lines and their relationship with ERα and HER2 receptors expression

**Fig. 1:**
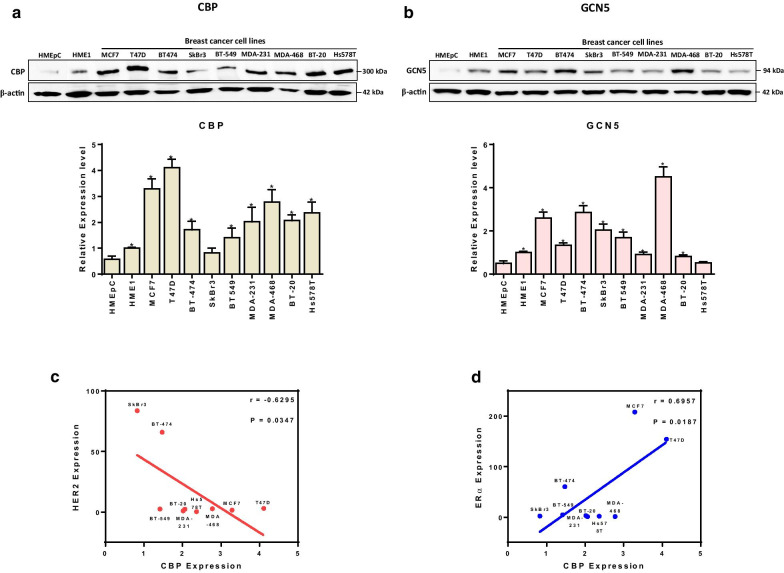
Baseline expression level of CBP and GCN5 in a panel of normal and cancer breast cells. Upper panel: Immunoblot images for **a** CBP and **b** GCN5 expression in normal cells (HMEpC and HME1) and in breast cancer cells (MCF7, T47D, BT474, SkBr3, BT-549, MDA-MB-231, MDA-MB-468, BT-20 and Hs578T). Lower panel: Graphs for relative expression level of CBP and GCN5 after normalization to β-actin. Full blots images are available in Additional file 1: Figure S4. Average present as mean ± SEM (*n* = 3). *p < 0.05 versus HMEpC, unpaired *t* test. **c**, **d** Correlation between the expression level of CBP and **c** the expression of HER2 or **d** ERα in nine breast cancer cell lines. Shown are “*r*” values (Pearson’s correlation coefficient) with the corresponding *p* values

Differential expression of CBP and GCN5 proteins in normal and malignant BC cells was investigated in an in-vitro model using a panel of nine BC cell lines with different ERα, PR and HER2 receptor status and two types of normal breast epithelial cells (Fig. 1a, b, Additional file 1: Fig. S1, Additional file 1: Table S1). Moreover, the relationship between baseline level of CBP and GCN5 and the status of ERα, PR, HER2 receptors expression of BC cell lines was also tested. The baseline level of CBP was higher in eight out of nine BC cell lines compared to the normal epithelial breast cells (Fig. 1a). Interestingly, there is a negative correlation between expression of HER2 and CBP (*r* = − 0.6295, *p* = 0.0347) (Fig. 1c, Additional file 1: Fig. S1). This is indicated by the high baseline level of CBP expression in the seven cell lines lacking HER2 overexpression (MCF7, T47D, BT-549, MDA-MB-231, MDA-468, BT-20 and HS578T) as well as by the low baseline level expression of CBP in the two cell lines overexpressing HER2 (BT-474 and SkBr3) (Fig. 1a). On the other hand, the expression of ERα is positively associated with a high baseline level of CBP (*r* = 0.6957, *p* = 0.0187) (Fig. 1d, Additional file 1: Fig. S1). This association is clear in cells not overexpressing HER2 (e.g., MCF7 and T47D); however, in ERα-positive, PR-positive cells which also overexpresses HER2, the effect of HER2 overexpression is predominant and the baseline level of CBP is low (e.g., BT-474 cells) (Fig. 1a, d). These results indicate a strong correlation between the receptor (ERα, PR and HER2) status of BC cells and the baseline level of CBP. It is noteworthy that the baseline level of CBP in most cell lines is time-(cell cycle phase) dependent. To cancel this effect, we seeded the same number of cells and we collected cells for protein extraction at the same time point for all tested cell lines. For the baseline level of GCN5, there is a general trend to be more expressed in BC cells than normal breast epithelial cells. Moreover and contrary to CBP, there seems no correlation between the baseline level of GCN5 and the expression of ERα, PR, or HER2 receptors in BC cells (Fig. 1b).

**Fig. 2:**
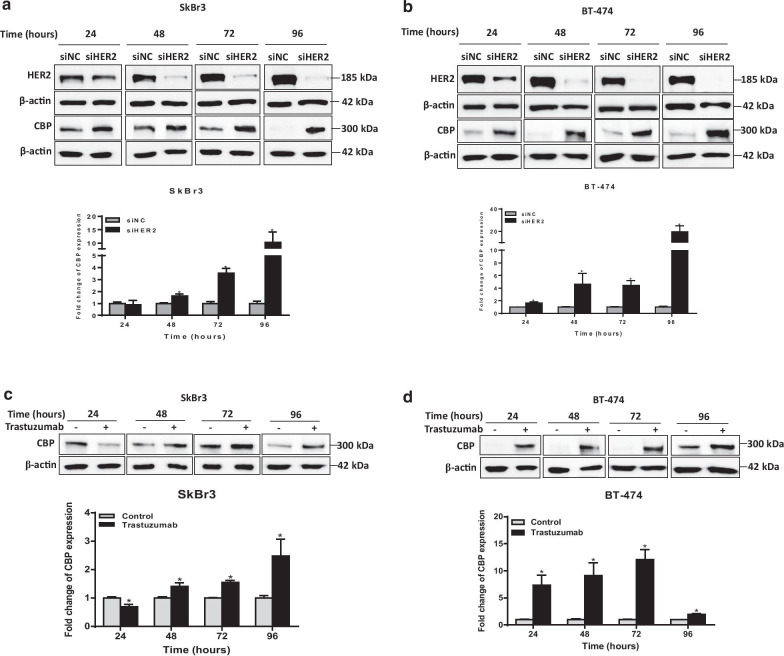
Biological or chemical inhibition of HER2 modulate the expression of CBP in HER2-overexpressing cells. **a**, **b** Upper panel: Representative western blot images for HER2 and CBP expression in **a** SkBr3 and **b** BT-474 cells transfected with negative control (NC) or HER2 siRNA for 24–96 h. Lower panel: Relative densitometric graphs of CBP expression after normalization to β-actin. Fold change of CBP expression was done over cells transfected with NC siRNA for each time point. **c**, **d** Upper panel: Images for immunoblots of CBP in **c** SkBr3 and **d** BT-474 cells treated with 10 μg/mL of HER2-specific humanized antibody Trastuzumab or with DMSO (control cells) for 24–96 h. Lower panel: Graphs for relative expression level of CBP after normalization to β-actin. Full blots images are available in Additional file 1: Fig. S5 and Additional file 1: Fig. S6. Average present as mean ± SEM (*n* = 3). *p < 0.05 vs untreated control, unpaired *t* test

**Fig. 3:**
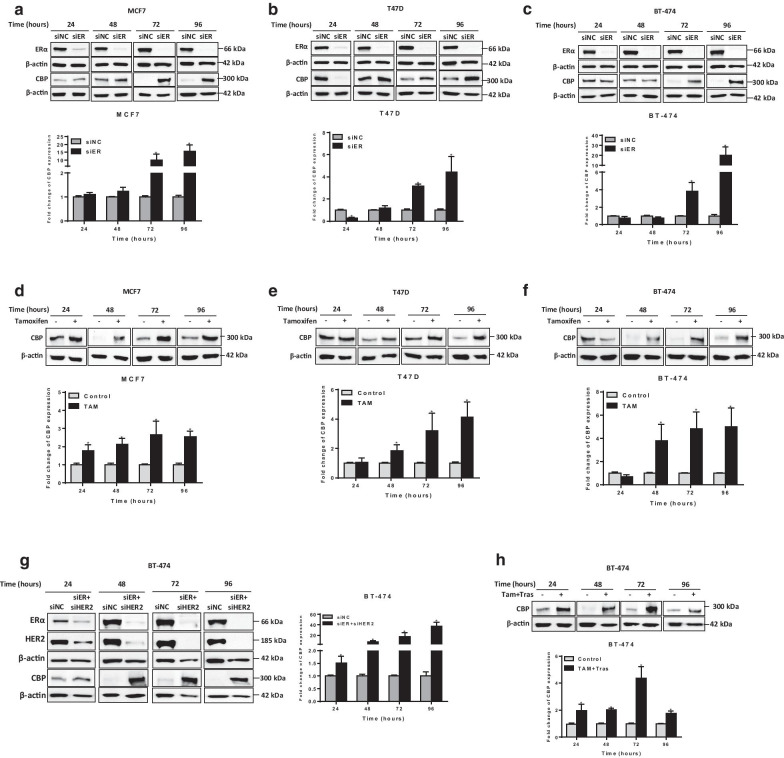
Biological or chemical inhibition of ERα or/and HER2 regulate CBP expression in ERα-positive cells. **a**–**c** Representative western blot analysis and relative bar graph quantification of CBP protein extracted from **a** MCF7, **b** T47D and **c** BT-474 cells transfected with negative control (NC) or ERα siRNA for 24–96 h. Fold change of CBP expression was done over cells transfected NC siRNA for each time point. **d-f** Upper panel: Representative western blot for CBP expression in **d** MCF7, **e** T47D and **f** BT-474 cells treated with 5 μM of the selective ER modulator Tamoxifen or with DMSO (control cells) for 24–96 h. Lower panel: Relative bar graph quantification of CBP protein after normalization to β-actin. **g** Western blot analysis of ERα, HER2 and CBP proteins in BT-474 cells transfected with negative control (NC) or ERα and HER2 siRNAs for 24–96 h. **h** Western blot analysis of CBP expression in BT-474 treated with Tamoxifen and Trastuzumab combination or with DMSO (control cells) for 24–96 h. The graphs show the densitometric quantification of CBP bands normalized to β-actin. Fold change of CBP expression was done over untreated control or cells transfected with NC siRNA for each time point. Full blots images are available in Additional file 1: Fig. S7, Additional file 1: Fig. S8 and Additional file 1: Fig. S9. Average present as mean ± SEM (*n* = 3). *p < 0.05 versus untreated control, unpaired *t* test

**Fig. 4:**
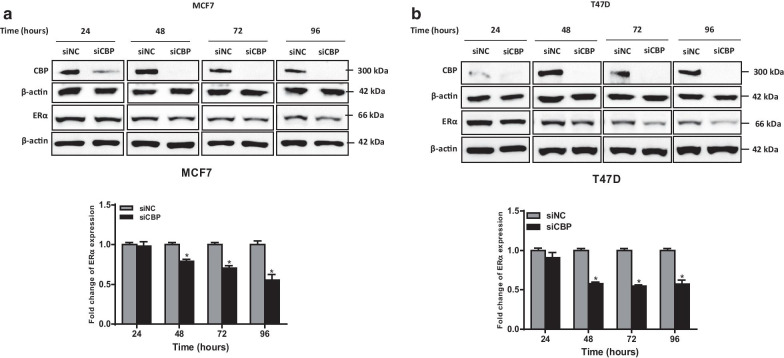
Effect of CBP downregulation by siRNA on the expression of ERα in MCF7 and T47D cells. **a**, **b** Western blot analysis of CBP and ERα proteins in **a** MCF7 and **b** T47D cells transfected with negative control (NC) or CBP siRNA for 24–96 h. The graphs show the relative amounts of ERα protein normalized to β-actin. Fold change of ERα expression was done over cells transfected with NC siRNA for each time point. Full blots images are available in Additional file 1: Fig. S10. Average present as mean ± SEM (*n* = 3). *p < 0.05 versus untreated control, unpaired *t* test

To investigate the nature of the crosstalk between CBP and the expression of ERα and HER2 receptors, we investigated the effect of the chemical and biological inhibition of ERα and HER2 on the expression of CBP in BC cells (Fig. 2). To cancel the effect of time-dependent expression of CBP, we used a separate control for each studied time point. Downregulation of HER2 by siRNA in HER2-overexpressing cell lines (SkBr3 and BT474) significantly increased the expression of CBP in both cell lines (Fig. 2a, b, Additional file 1: Fig. S2a, b). Particularly, the highest level of increase in CBP (more than 10 folds) was observed at 96 h of HER2 downregulation. The same results were obtained upon chemical inhibition of HER2 by trastuzumab; however, the increase in the level of CBP was observed at earlier time points (24 and 48 h) (Fig. 2c, d). These results confirm the negative feedback between HER2 and CBP. Similarly, the biological and the chemical inhibition of ER-α resulted in overexpression of CBP in three ERα-positive BC cell lines (MCF7, T47D and BT-474) (Fig. 3). Again, the biological inhibition of ERα resulted in late (at 72 and 96 h) overexpression of CBP in the three cell lines (Fig. 3a–c, Additional file 1: Fig. S2a–e), whereas the effect of chemical inhibition of ERα by tamoxifen was observed at earlier time points (starting 24 h) in all cell lines (Fig. 3d–f). In addition, the downregulation or chemical inhibition of both receptors (ER-α and HER2) in the triple-positive BC cell line (BT-474) resulted in the upregulation of CBP level (Fig. 3g, h, Additional file 1: Fig. S2f, g). To check whether CBP is acting upstream or downstream of ERα, we downregulated the expression of CBP in MCF7 and T47D cells (expressing a high baseline level of CBP) and we measured the expression of ERα (Fig. 4, Additional file 1: Fig. S3). Figure 4 shows that the downregulation of CBP resulted in a reduction in the level of ERα in both cell lines. Collectively, these results indicate a strong association between CBP and the expression of ERα and HER2 in which a clear direct relation exists between CBP and ERα. On the other hand, the relationship between CBP and HER2 expression is not straight forward like ERα.

### Expression of CBP and GCN5 in clinical breast tissue samples

**Fig. 5:**
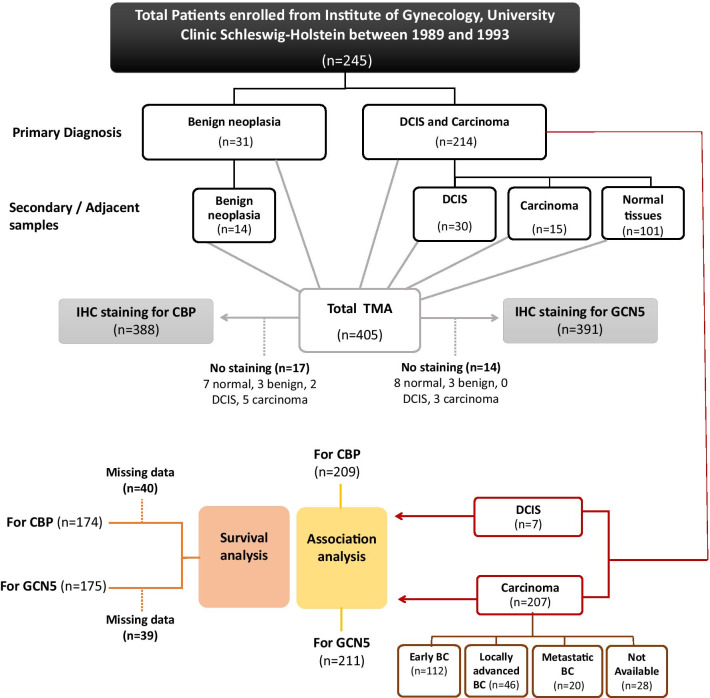
Consort flow diagram showing patient’s inclusion in study association and survival analysis. The other missing data for histopathological parameters of the included patients are mentioned in Table 1

**Fig. 6:**
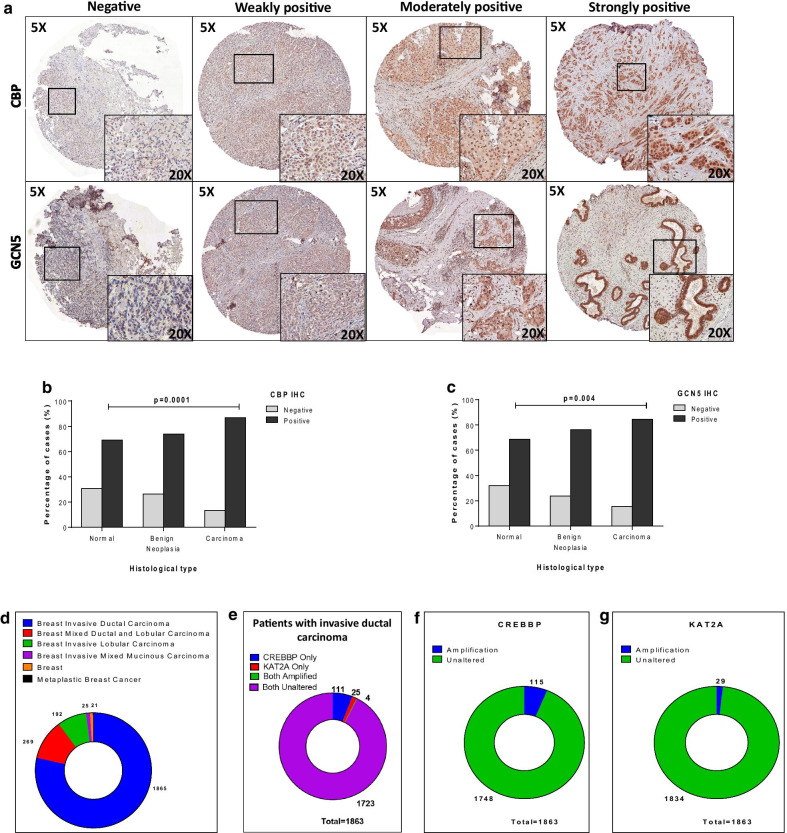
Expression of CBP and GCN5 in clinical breast tumors. **a** 388 and 391 samples were analyzed by immunohistochemistry for the expression of CBP and GCN5, respectively. Samples were scored using immunoreactivity scoring system (IRS) and designated as negative (IRS = 0–3), weakly positive (IRS = 4–5), moderately positive (IRS = 6–7) and strongly positive (IRS = 8–9). **b, c** Immunohistochemistry analysis of **b** CBP and **c** GCN5 expressions in normal, benign lesion and breast carcinoma tissues of breast cancer patients. *p < 0.05, Pearson Chi-Square test. **d** Publicly available dataset of breast cancer patients were acquired from METABRIC database for different types of breast cancer. **e** The patients with ductal carcinoma (*n* = 1863) were filtrated for genetic alterations analysis of *CREBBP* and *KAT2A* genes. **f**, **g** Pie Charts for the amplification of **f** *CREBBP* gene and **g** *KAT2A* gene in ductal carcinoma patients from METABRIC database

The expression of CBP and GCN5 was investigated in human BC tissues and normal breast tissues using the immunohistochemical (IHC) approach as described in consort diagram (Fig. 5). The characteristics of the studied BC patients’ specimens are presented in Table 1. The majority of the included BC cases were diagnosed primarily as invasive breast carcinoma (IBC) of no special type (NST) with/without an associated in situ component. IBC NST constitutes the majority of the histological subtypes of BC [36]. Few cases were reported as ductal carcinoma in situ where no invasion component could be identified (Additional file 1: Table S2). Representative images of negative and positive expression of CBP and GCN5 are shown in Fig. 6a. The subcellular distribution of CBP protein showed its localization in the nuclei of normal and BC cells and less frequently detected in the cytoplasm. On the other hand, GCN5 protein is distributed in both nuclei and cytoplasm (Fig. 6a). IHC staining showed CBP protein to be significantly more expressed in breast carcinoma samples both the “invasive and the in situ components” samples (No. of positive cases/total: 217/252) compared with benign neoplasia samples (No. of positive cases/total: 31/42), and normal breast samples (No. of positive cases/total: 65/94) (*p* = 0.0001) (Fig. 6b). Similarly, GCN5 protein expression is upregulated in breast carcinoma sections ((No. of positive cases/total: 215/256) compared with benign neoplasia (No. of positive cases/total: 32/42), and normal breast sections (No. of positive cases/total: 64/93) (*p* = 0.004) (Fig. 6c). To get more conclusive insight into the expression of *CREBBP* (For CBP) and *KAT2A* (For GCN5) genes in BC patients, the publicly available database (https://www.cbioportal.org/) was used to download the clinical, pathological and omics data for each patient in the dataset. BC dataset (METABRIC, Nature 2012 and Nat Commun 2016) was used as it includes 2,509 BC patients [37] (Fig. 6d). Since we are interested in ductal carcinoma, we selected the ductal carcinoma cases only for further analysis. Totally, 1863 samples were filtered and searched for alteration in *CREBBP* and *KAT2A* genes in terms of mutation, amplification, or altered gene expression (Fig. 6e). *CREBBP* gene is altered in 115 (6%) of 1863 queried samples, and all the alterations were of the amplification type (Fig. 6f). On the other hand, *KAT2A* was amplified in 29 (2%) of queried patients (Fig. 6g). These results indicated that CBP and GCN5 are overexpressed in breast tumors as well as their genes were amplified in some BC patients.

**Table 1:** Clinical characteristics of the patients and histopathological parameters of the tumors of breast cancer (DCIS and carcinoma) patients (primary diagnosis, n = 214)

Variables	N (%)	No. of missing data (%)	Variables	N (%)	No. of missing data (%)
*Age at diagnosis (years)*		0 (0.0)	*ER status*^a^		9 (4.2)
≤ 40	15 (7.0)		Negative	78 (38.0)	
41–70	127 (59.4)		Positive	127 (62.0)	
> 70	72 (33.6)				
*Tumor stage*		29 (13.6)	*PR status*^a^		13 (6.1)
pTis	6 (3.2)		Negative	75 (37.3)	
pT1	51 (27.6)		Positive	126 (62.7)	
pT2	80 (43.2)		*HER2 overexpression*		7 (3.3)
pT3	8 (4.3)		Negative	184 (88.9)	
pT4	40 (21.6)		Positive (> 30%)	23 (11.1)	
*Histological grade*		21 (9.8)	*Ki67 status*		2 (0.9)
G1	8 (4.1)		≤ 20%	188 (88.7)	
G2	149 (77.2)		> 20%	24 (11.3)	
G3	36 (18.7)				
*Lymph node metastasis*		45 (21.0)	*Subtypes *[58]		41 (19.2)
Negative	84 (49.7)		Luminal A	86 (49.7)	
Positive	85 (50.3)		Luminal B HER2−	23 (13.3)	
			Luminal B HER2+	10 (5.8)	
			HER2-overexpressed	9 (5.2)	
			Triple negative	45 (26.0)	

^a^The sample is considered ER/PR negative if < 10% of tumor cell nuclei are immunoreactive [59]

### The level of CBP expression correlates with ERα and PR protein expression in patient samples

**Fig. 7:**
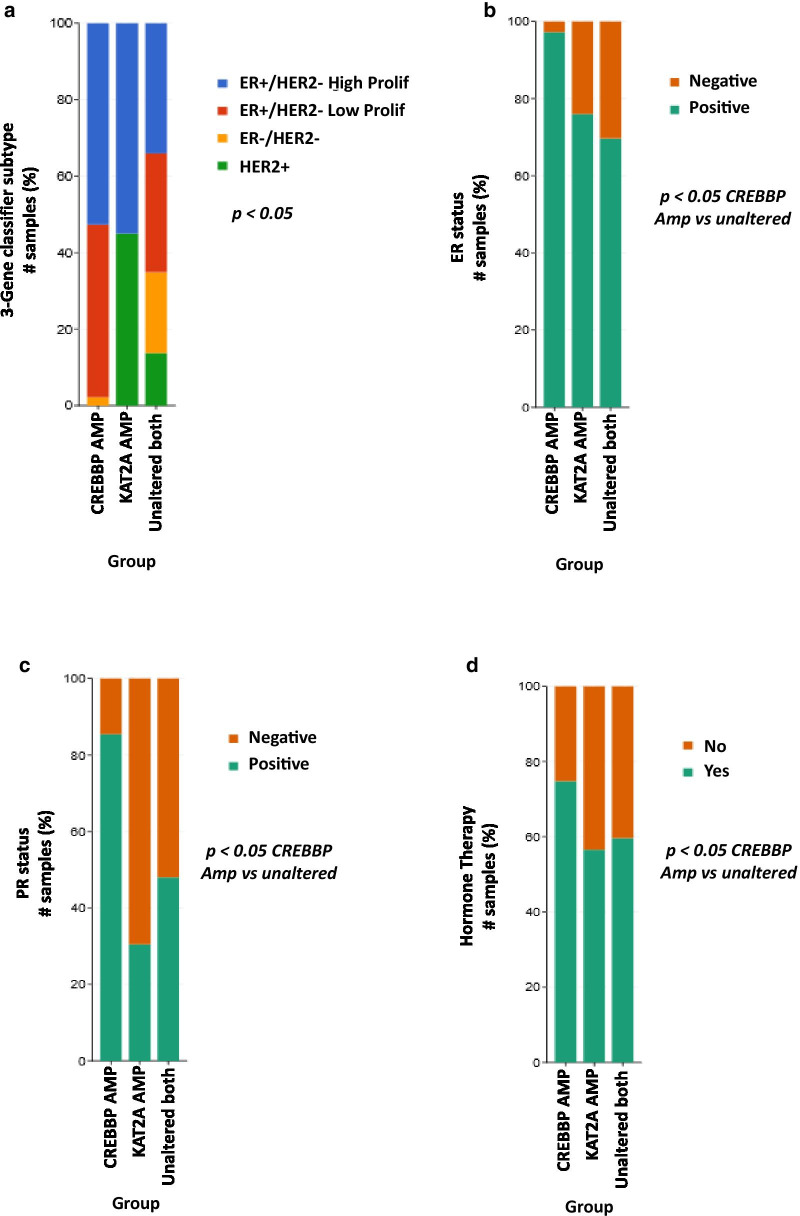
*CREBBP* and *KAT2A* gene amplification in breast cancer cases with different ERα and PR status. **a** Bars depict the association of *CREBBP* and *KAT2A* genes amplification (AMP) across different breast cancer subtypes (Luminal B: ERα+/HER2+-high proliferation, Luminal A: ERα+/HER2+-low proliferation, Triple negative: ERα−/HER2− and HER2-overexpressed: HER2+). **b**, **c** The amplification status of *CREBBP* and *KAT2A* genes are depicted for **b** ERα+ (green) and ERα− (orange) samples and for **c** PR+ (green) and PR− (orange) samples. **d** The distribution of breast cancer patients who received hormonal therapy according to their *CREBBP* and *KAT2A* gene amplification status. *p* < 0.05 is statistically significant

Next, we investigated the expression of CBP and GCN5 with respect to BC subtypes in our patients’ samples (Table 2). The results showed that CBP protein is more frequently expressed in luminal A, luminal B HER2-negative and luminal B HER2-positive compared with other molecular (No. of positive cases/total: 82/85, 20/25 and 9/9, respectively, *p* = 0.0001). On the other hand, no significant variation in the level of GCN5 is observed between different BC subtypes (*p* = 0.166) (Table 2). The results of the public data of BC patients demonstrated the persistent amplification of *CREBBP* gene in luminal A, luminal B and triple-negative subtypes, whereas no *CREBBP* amplification was observed in HER2 positive subtypes (Fig. 7a). This supports the same finding in our cohort of patients’ samples. However, *KAT2A* gene was observed to be significantly amplified in luminal B and HER2-overexpressing subtypes (Fig. 7a).

**Table 2:** Expression of CBP and GCN5 in tissue samples from breast cancer patients with different subtypes by IHC

Group [58]	CBP expression			GCN5 expression		
	Negative (%)^a^	Positive (%)^b^	*p* value^c^	Negative (%)^a^	Positive (%)^b^	*p* value^c^
Luminal A	3 (3.5)	82 (96.5)	0.0001	9 (10.7)	75 (89.3)	0.166
Luminal B HER2-	3 (13.0)	20 (87.0)		3 (13.6)	19 (86.4)	
Luminal B HER2+	0 (0)	9 (100)		1 (11.1)	8 (88.9)	
HER2−overexpressed	3 (37.5)	5 (62.5)		1 (11.1)	8 (88.9)	
Triple negative	11 (25.6)	32 (74.4)		8 (17.8)	37 (82.2)	
Total	168			169		
No. of missing data (%)	41 (19.6)			42 (19.9)		

^a^Negative: IRS 0 to 5

^b^Positive: IRS 6 to 9

^c^Chi-square test

Investigating the expression level of CBP and GCN5 in BC tissue samples with different receptors status revealed a significantly high level of CBP expression in the ERα-positive and PR-positive BC compared to ERα- and PR-negative tissue samples (*p* = 0.0001 and *p* = 0.0001, respectively) (Table 3), whereas the expression level of GCN5 did not show a significant correlation with the status of ERα or PR hormone receptors (*p* = 0.213 and *p* = 0.541, respectively) (Table 3). Additionally, no significant correlation is found between the positive expression of both HATs (CBP and GCN5) with HER2 overexpression nor ki-67 status. In line with the results of tissue samples, data from publicly available BC databases showed a strong correlation between CBP amplification and ERα and PR status, whereby ERα-positive and PR-positive samples were associated with *CREBBP* amplification more than their corresponding ERα- and PR-negative samples (Fig. 7b, c). On the other hand, no correlation was observed between *KAT2A* gene amplification and ERα or PR status (Fig. 7b, c).

**Table 3:** Correlation between CBP and GCN5 expression and the status of ER, PR, HER2 and ki-67 in tissue samples from breast cancer patients

Molecular subtypes	Total	No. of missing data (%)	CBP expression			Total	No. of missing data (%)	GCN5 expression		
			Negative (%)^a^	Positive (%)^b^	*p* value^c^			Negative (%)^a^	Positive (%)^b^	*p* value^c^
*ER status*										
Negative	75		17 (22.7)	58 (77.3)	0.0001	78		14 (17.9)	64 (82.1)	0.213
Positive	124		6 (4.8)	118 (95.2)		123		14 (11.4)	109 (88.6)	
Total	199	10 (4.8)				201	10 (4.7)			
*PR status*										
Negative	71		17 (23.9)	54 (76.1)	0.0001	74		13 (17.6)	61 (82.4)	0.541
Positive	125		7 (5.6)	118 (94.4)		123		17 (13.8)	106 (86.2)	
Total	196	13 (6.2)				197	14 (6.6)			
*HER2*										
Negative	180		22 (12.2)	158 (87.8)	1.000	181		26 (14.4)	155 (85.6)	0.552
Positive (> 30%)	21		3 (14.3)	18 (85.7)		22		2 (9.1)	20 (90.9)	
Total	201	8 (3.8)				203	8 (3.8)			
*Ki-67 status*										
≤ 20%	183		22 (12.0)	161 (88.0)	0.354	184		30 (16.3)	154 (83.7)	0.094
> 20%	24		4 (16.7)	20 (83.3)		24		1 (4.2)	23 (95.8)	
Total	207	2 (1.0)				208	3 (1.4)			

^a^Negative: IRS 0 to 5

^b^Positive: IRS 6 to 9

^c^Chi-square test

The strong association between CBP and ERα was further confirmed from the publicly available BC datasets of ERα-positive BC patients who received hormonal therapy. Correlation analysis showed that a high percentage of BC patients have amplification in *CREBBP* gene after receiving the hormonal therapy (Fig. 7d). This supports the in-vitro results of a positive correlation between CBP and ERα.

### Relationship between CBP and GCN5 expression and clinicopathological features and survival of BC patients

To check the clinical significance of CBP and GCN5 expression in BC patients, we investigated the clinical characteristics of the patients and histopathological parameters of the tumors of our BC patient cohort, e.g., age at diagnosis, tumor type and its histological grade, lymph node status and the TNM stage of breast carcinomas as well as the survival of the patients. However, no significant associations were found between the CBP or GCN5 expression and any of the studied clinicopathological parameters (*p* > 0.05) (Additional file 1: Table S3).

**Fig. 8:**
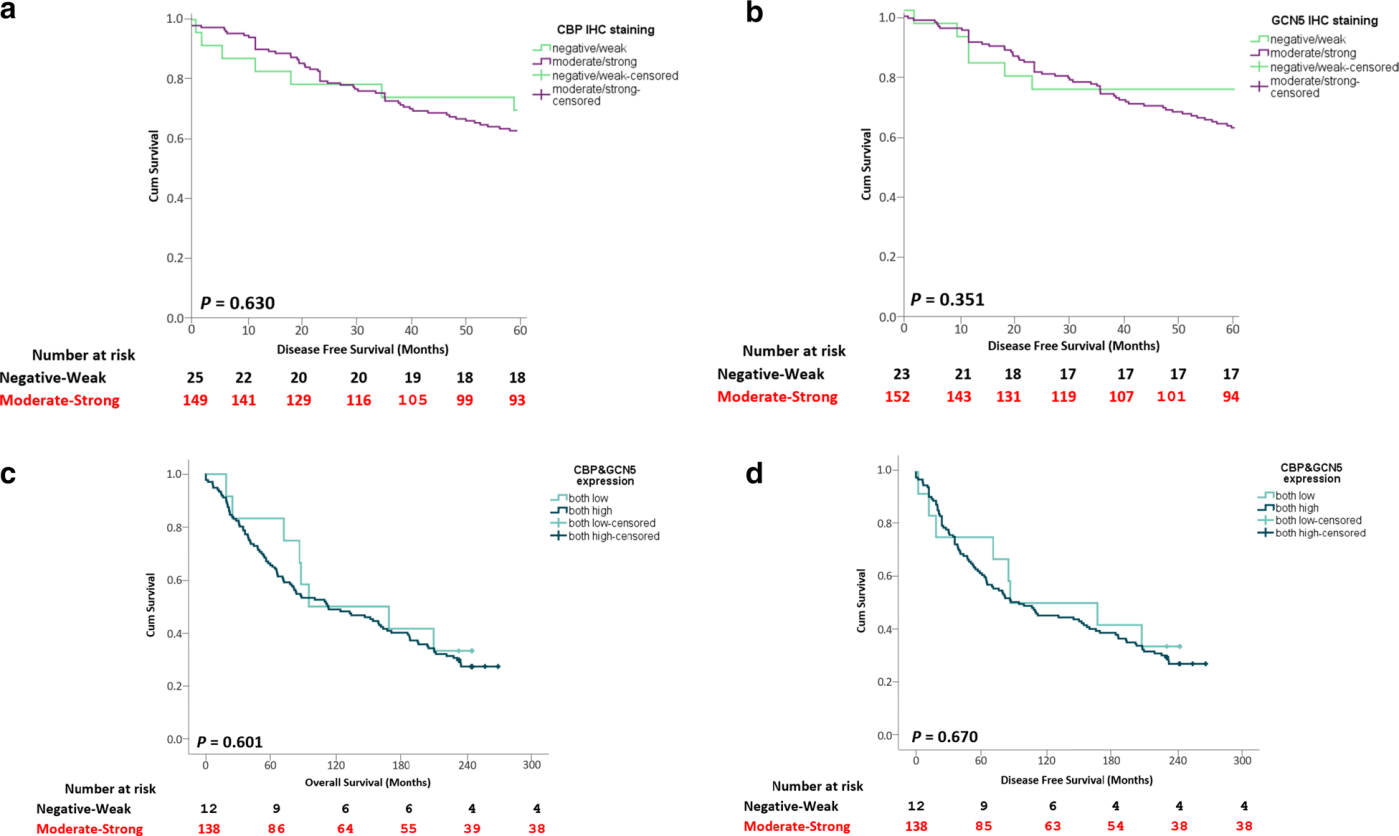

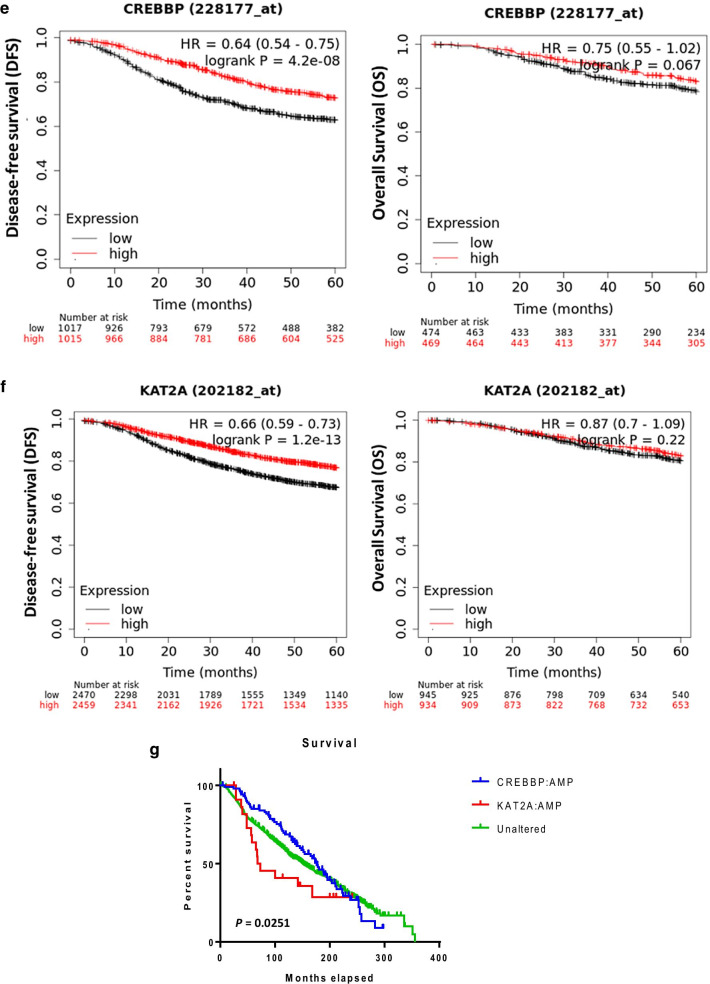
Correlation of CBP and GCN5 expression with breast cancer patients’ survival. **a**, **b** Kaplan–Meier survival curves of disease-free survival for **a** CBP and **b** GCN5 expression. **c**, **d** Representative **c** overall survival and **d** disease-free survival curves for both CBP and GCN5 expression. **e**, **f** 5-year disease-free survival and overall survival analyses for **e** *CREBBP* and **f** *KAT2A* mRNA expression (http://kmplot.com/). **g** Overall survival curves for *CREBBP* and *KAT2A* amplification in breast cancer patients. Significant differences were calculated using the log-rank test. *p* < 0.05 is statistically significant

The influence of CBP and GCN5 expressions on the overall survival (OS) or disease-free survival (DFS) of BC patients was investigated using a log-rank test (Fig. 8). The analysis showed no significant correlation between CBP or GCN5 expression and the 5-year DFS (*p* = 0.630 and 0.351 for CBP and GCN5, respectively) (Fig. 8a, b). Similarly, no clear impact was found for high CBP nor high GCN5 expression on the OS and the DFS of BC patients (*p* = 0.601, 0.670 for OS and DFS, respectively) (Fig. 8c, d). Also, the level of expression of the two proteins (CBP and GCN) did not correlate significantly to the DFS in the different BC types (Additional file 1: Fig. S11, Additional file 1: Fig. S12). However, the analysis of the publicly available data of *CREBBP* and *KAT2A* genes expression in a larger number of patients showed significant correlation with regard to the 5-year DFS (Fig. 8e–g).

Kaplan–Meier Plotter online tool (http://kmplot.com/) was used to examine the survival in BC patients. Patients were divided into two groups (low and high expression) according to the mRNA expression of the given genes. Patients with high *CREBBP* gene expression had better 5-year DFS rates than the low gene expression group (*p* = 0.0018) (Fig. 8e). Similarly, the high *KAT2A* gene expression correlated significantly with better 5-year DFS of BC patients (*p* < 0.00001) (Fig. 8f). Analyzing the OS showed significant differences between groups of patients with amplified *CREBBP*, *KAT2A* or no amplification of both genes (*p* = 0.025), whereby *CREBBP* amplification is associated with better OS and *KAT2A* gene amplification with worse OS (Fig. 8g).

## Discussion

Although great advances have been made in the management of early stages BC, a considerable fraction of patients might progress into metastatic BC [38]. Despite the different therapeutic options available for the treatment of metastatic BC (such as endocrine therapy, tyrosine kinase inhibitors, growth factors antagonists, PARP inhibitors and conventional chemotherapy), advanced metastatic BC is considered incurable [39].

Histone acetyltransferases (HAT) regulate many cellular processes by modifying the acetylation status of histone and non-histone proteins and by acting as transcriptional co-activators [40]. Thus, reporting aberrant activity and/or expression of different HATs in many diseases including cancer is not surprising. The main aim of this study is to investigate the role of two HATs: namely CBP and GCN5 as diagnostic or prognostic markers in BC. Moreover, we aim at studying the relationship between these two HATs and the expression of ERα, PR and HER2 receptors in BC. This might help in more understanding of the pathogenesis of BC and hence developing new diagnostic and prognostic markers and new therapeutic targets.

The loss of CBP was reported previously to be associated with the initiation of basal-type BC, which is known to be aggressive, resistant to anti-cancer drugs and with high mortality rate. This was attributed to the inability of breast cells to execute apoptosis upon loss of CBP [41]. In another study, CBP was found to be highly expressed in triple negative BC patients, an aggressive BC subtype and its overexpression correlated to positive lymph node metastasis but not with the overall survival [42]. The overexpression of the CBP paralog, p300, in breast carcinoma was previously reported, and it was evaluated as an independent biomarker for poor prognosis of BC patients [43]. Our results revealed high expression and/or amplification of CBP and GCN5 in BC compared to benign neoplasia samples and normal breast samples. Importantly, high CBP expression or amplification was correlated with the positive status of ERα and PR receptors and it was displayed more in Luminal A and Luminal B subtypes. This may reflect the value of CBP and GCN5 as diagnostic markers in BC. The higher degree of protein expression in carcinomas in the TMAs of our cohort compared to the relatively low number of genetic alterations in publicly available datasets may be explained by the fact that in some cases, non-detectable levels of gene expression had no effect on the levels of the detected protein expression, suggesting fast translation in the case of a short half-life or efficient translation from a small amount of mRNA in the case of a low level of mRNA. This is reported in the literature [44]. Moreover, the discrepancy between our results and the publicly available data regarding the correlation between the level of the two proteins and the patients’ DFS or OS may be due to the fact that the survival analysis has been done on the proteins at different levels of gene expression (i.e., mRNA and protein) and each level could be differentially regulated, which might result in this variation. In addition, although datasets provide a valuable resource to test hypotheses for individual genes/signatures, there are variations in terms of size, patient characteristics and molecular composition of datasets and they do not necessarily reflect the studied cohort of BC patients. This is reported in the literature [45]. However, this indicates the need for more studies to test the value of these two proteins as prognostic markers in BC. Previously p300, a paralog of CBP, was reported as a bad prognostic marker in BC [43]. Although CBP and p300 have overlapping functions, pieces of evidences exist for unique roles and pathways [46].

The high level of CBP and GCN5 in BC cells/tissues might be a cause or a consequence of the malignant transformation. Their high level may enhance malignant transformation by increasing the activity of the growth-promoting genes (oncogenes) through enhanced acetylation of their promoter areas or through stimulating their activity by acting as transcriptional coactivators. This concept contradicts with the report of Dietze et al. who reported that loss of CBP in human mammary epithelial cells is associated with the inability of cells to execute apoptosis and increases the risk of basal-type BC [47]. On the other hand, a malignant transformation may increase the expression of CBP and GCN5 to enhance the expression of genes involved in processes such as angiogenesis, DNA repair, invasion and migration aiming to support the high level of division of malignant cells or to help them to accommodate for cellular stress. This point needs more deep investigations to understand the role of CBP and GCN5 in breast carcinogenesis and whether they are involved in the early stages of carcinogenesis or they are needed for the late events of building up a malignant tumor mass.

We also report for the first time the existence of a reciprocal relationship between CBP and ERα and CBP and HER2. CBP is an established transcriptional coactivator of ERα; therefore, downregulation/chemical inhibition of ERα reduces the consumption of CBP and increases its free level. On the other hand, the downregulation of CBP might reduce the level of ERα by one or two ways; (1) reduction of the acetylation of the ERα gene promoter area and the subsequent reduction of ERα mRNA transcription, or (2) reduced level of CBP which is a transcriptional coactivator of ERα results in a reduction of the transcriptional activating activity of ERα and less ability to bind DNA with subsequent enhanced ERα degradation. Another hypothesis is that the CBP is controlling the expression of ERα (i.e., CBP acts upstream of ERα); therefore, when the level of CBP is low, the level of ERα will be low (as in Fig. 1d) and when ERα is inhibited (biologically or pharmacologically), the level of CBP will be increased to compensate (which is shown in Fig. 3). This hypothesis is confirmed in Fig. 4, whereby downregulation of CBP resulted in downregulating the expression of ERα. A similar relationship between ERα and CBP was reported previously whereby ligand-activated ERα induced reduction of the histone acetyltransferase activity of CBP [48]. Also, CBP is involved in estrogen receptor signaling through inducing its acetylation and enhancing its transcriptional- and DNA binding activities [49]. In addition, the public data analysis in our current study showed enhanced *CREBBP* gene amplification in tumor specimens from BC patients who received hormonal therapy. These clinical analyses support our in-vitro findings for the crosstalk between CBP and ERα. On the other hand, ER positivity was massively reported to be associated with better prognosis and survival outcomes in BC patients [50–53]. The positive correlation between CBP and ERα in the BC patients as indicated in this study proposes that the prognostic significance of CBP in BC could be similar to ERα and introducing CBP as a favorable prognostic biomarker. The increased expression of CBP upon HER2 downregulation by siRNA or inhibition by Trastuzumab suggests a negative effect of HER2 on CBP expression. This may be due to: (1) CBP is involved in HER2 signaling and inhibition of HER2 conserves the CBP and hence increases its level, (2) CBP is involved in HER2 expression by acetylating the promoter area of its gene and inhibition of HER2 results in an increased level of CBP to compensate, or (3) HER2 inhibition induces cellular stress and/or DNA damage and CBP level is enhanced as a response to this cellular stress. In this context, a previous investigation showed that the RAS-PI3K-AKT, a downstream pathway of HER2, targets CBP via the MDM2-dependent degradation [54]. Since ERα and HER2 signaling pathways are critical for BC progression and therapy, our report of crosstalk between CBP, ERα and HER2 emphasizes the role of CBP in BC. However, more work would be needed to understand the functional interaction between CBP, ERα and HER2 in BC.

## Conclusions

In conclusion, we report the overexpression of CBP and GCN5 in BC cells/tissues more than the normal ones. The relationship between CBP and GCN5 expression and patients DFS or OS requires more investigations. Interestingly, a bidirectional crosstalk exists between CBP, ERα and HER2, which suggests the contribution of CBP in BC pathogenesis. Our results present the CBP as a potential diagnostic marker and a therapeutic target in hormone receptor-positive BC.

## Methods

### Cell lines

The BC cell lines were purchased from ATCC (VA, USA) (Additional file 1: Table S1). MCF7, T47D, BT-549 and MDA-MB-231 were cultured in RPMI (Sigma Aldrich, USA), while BT-474, SkBr3, MDA-MB-468, BT-20 and Hs578T were cultured in DMEM supplemented with 10% fetal bovine serum and 1% penicillin/streptomycin (Sigma Aldrich, USA). The immortalized human mammary epithelial cells hTERT-HME1 (ATCC, USA) were grown in DMEM/F-12 mixture medium supplemented with 10% fetal bovine serum and 1% penicillin/streptomycin (Sigma Aldrich, USA). The human mammary epithelial cells HMEpC were purchased from cell applications (CA, USA) and maintained in defined mammary epithelial cell medium provided by the company (Cell applications, USA).

### Drug treatment

The MCF7, T47D, SkBr3 and BT-474 cells were seeded in T25 cm^2^ flasks at a density of 800,000–200,000 cells/flask. In the next day, the MCF7, T47D and BT-474 cells were treated with 5 µM of Tamoxifen (Sigma-Aldrich, USA), SkBr3 and BT-474 cells were treated with 10 µ/mL of Trastuzumab (Mylan, US), and BT-474 cells were treated with Tamoxifen and Trastuzumab combination. The cells were harvested after 24, 48, 72 and 96 h of treatment for protein extraction.

### siRNA transfection

The MCF7, T47D, SkBr3 and BT-474 cells were seeded in 6-well plate at a density of 400,000–300,000 cells/well in antibiotic-free medium. In the next day, cells were transfected with siRNA using lipofectamine RNAiMAX reagent (Thermo Fisher scientific, USA) following manufacturer’s recommendations. The cells were transfected with 50 nM of ON-TARGET plus SMART pool siRNA against *ESR1* (L-003401-00-0005), *ERBB2* (L-003126-000005) and *CREBBP* (L-003477-000005) (Dharmacon, USA). Non-targeting siRNA (D-001810-1005) was used as negative control. The cells were harvested after 24, 48, 72 and 96 h of transfection for protein extraction.

### Protein extraction and western blot

Analysis of the protein expression was performed as described previously [55]. Briefly, total cell lysates from breast cell lines were prepared in lysis buffer (20% SDS, glycerol, 1 M Tris (pH 6.8)) containing protease inhibitor cocktail (Sigma-Aldrich, USA). An equal amount of proteins (10 µg) were loaded and separated in 8% SDS-PAGE or 3–8% Tris–acetate gel (for CBP only) and then transferred into nitrocellulose membrane (Sigma-Aldrich, USA). Followed by immunoblotting with rabbit monoclonal primary antibodies against CBP (#7389), GCN5 (#3305), ERα (#13258) and HER2 (#4290) (Cell Signaling Technology, USA) at dilution 1:1000 and mouse monoclonal antibody against β-actin (#A5441) at dilution 1:2000 (Sigma-Aldrich, USA), then with secondary anti-rabbit IgG, HRP-linked antibody and anti-mouse IgG, HRP-linked antibody (Cell Signaling Technology, USA). The detection of membrane was carried out by the ECL method (Biorad, USA) and developed using ChemiDoc™ imaging system (Biorad, USA). The band quantification was carried out using Image Lab™ software (Biorad, California, USA) with β-actin as loading control.

### Breast cancer tissue samples

For clinical validation of differentially expressed proteins, breast carcinomas and adjacent normal mucosa were randomly selected from patients that were part of a patient cohort undergoing surgery for breast malignancy at the Institute of Gynecology, University Clinic Schleswig–Holstein, Campus Lübeck, Germany, between 1989 and 1993. All samples were archived in the Institute of Pathology at the University Clinic Schleswig–Holstein, Campus Lübeck, Germany. This study encompassed 405 formalin-fixed paraffin-embedded (FFPE) breast tissue samples which were obtained from 245 patients primarily diagnosed with invasive breast carcinoma, carcinoma in situ or benign neoplasia. In a subset of patients, supplementary samples were collected from the tissue adjacent to the sample of the primary histological diagnosis. These supplementary samples have been used for comparison between the histological groups. A total of 101 normal tissue samples were collected from the apparently normal tissue adjacent to the tumor mass. The FFPE samples were obtained at the Section of Translational Surgical Oncology and Biobanking, Department of Surgery, University Medical Center Schleswig–Holstein-Lübeck-Germany, adhering to the guidelines of the local ethical review board (#08-012). For association and survival analysis, only samples of the breast cancer taken for primary diagnosis were evaluated. The clinical characteristics of the patients and histopathological parameters of the tumors of the study cohort are detailed in Table 1 and Additional file 1: Table S2.

### Tissue microarray construction

Tissue microarray was constructed as described previously [56]. Tissue cores of 1.5 mm diameter were punched from selected regions of FFPE donor tissue blocks and embedded into recipient paraffin block using semi-automated arrayer (TMArrayer; Pathology Devices, MD, USA).

### Immunohistochemistry staining

The immunohistochemistry for CBP and GCN5 was performed manually. Deparaffinization of the unstained sections was carried out by xylene followed by rehydration in a series of ethanol. Subsequently, the antigen retrieval was carried out using citrate buffer and heated for 5 min at 900 W followed by heating for 10 min at 750 W for two times. After cooling down, the slides were washed three times with PBS and the endogenous peroxidase was blocked for 10 min by 3% hydrogen peroxide. The slides were subsequently washed three times with PBS and blocked with goat serum for 45 min, followed by incubation with CBP (# sc-7300) or GCN5 (# sc-365321) primary antibody at dilution 1:100 (Santa Cruz Biotechnology, USA) at 4 °C overnight. On the next day, the slides were washed with PBS and incubated with rabbit secondary antibody labeled with biotin at dilution 1:50 for 30 min (Dako, USA) followed by the addition of diaminobenzidine substrate (Dako, USA) in combination with avidin–peroxidase complex solution. Finally, the slides were counterstained with hematoxylin, covered with aquatex and scanned by a digital microscope (Pannoramic DESK, 3D Histech, Budapest, Hungary).

### Immunohistochemistry interpretation

Immunopositivity of CBP and GCN5 was assessed semi-quantitatively by two independent observers to confirm the reproducibility of the results. The whole TMA cores in each tumor and non-tumor breast tissue were evaluated. The percentage of positively stained tumor cells (PP) and the staining intensity (SI) were determined. The immunoreactive score (IRS) was as follows: IRS = SI × PP, for each sample, as previously described [57]. The intensity was scored as follows: 0: No staining, 1: weakly positive, 2: moderately positive and 3: strongly positive. The percentage of positively stained cells was given the following scores: score 0: 0–1% positive cells, score 1: 2–20% positive cells, score 2: 21–50% positive cells and score 3: 51–100% positive cells. The IRS score thus ranged from 0 to 9, designated as negative for a score of 0 to 3, weakly positive for a score of 4 or 5, moderately positive for a score of 6 or 7 and strongly positive for a score of 8 or 9. Localization of the positivity was also determined: nuclear, cytoplasmic, or mixed.

### Publicly available cancer genomics and patients’ data

In order to explore the expression and the clinical significance of *CREBBP* (CBP) and *KAT2A* (GCN5) genes in BC patients, the publicly available database (https://www.cbioportal.org/) was used to extract the clinical, pathological and omics data for each patient in the dataset. BC dataset (METABRIC, Nature 2012 and Nat Community 2016) was used, it includes 2509 BC patients [37]. Invasive breast carcinoma cases were selected for further analysis. In order to evaluate the prognostic value of CREBBP and KAT2A mRNA expression, Kaplan–Meier Plotter online tool (http://kmplot.com/) was used to investigate the OS in BC patients. Patients were divided into two groups (low and high expression) according to the mRNA expression of the given genes.

### Statistical analysis

GraphPad Prism 6 (GraphPad Software, USA) and SPSS statistics (IBM corporation, USA) were used for statistical analysis. For in-vitro experiments, the results are expressed as the means ± SEM of at least three independent experiments and the unpaired student t-test was used for statistical analysis. Association between CBP and GCN5 expression and clinical characteristics of the patients and histopathological parameters of the tumors were examined using the Pearson chi-square test. Kaplan–Meier analysis was used to generate survival curves. Log-rank tests were used to assess the differences between groups in overall survival (OS) and disease-free survival. *p* value < 0.05 was considered as statistically significant.

## Supplementary Information

**Additional file 1.** **Supplementary Tables: Table S1.** Molecular subtypes of breast cancer cell lines. **Table S2.** Histopathological features of tissue microarray samples. **Table S3.** Clinical-pathological parameters and CBP & GCN5 expression in DCIS and breast carcinoma cases. **Supplementary Figures: Fig. S1.** Baseline expression level of ER and HER2 in a panel of normal cancer breast cells. **Fig. S2.** Efficiency of transfection kinetics for a, b HER2 siRNA, c-e ER siRNA and f, g both ER and HER2 siRNAs. **Fig. S3.** Efficiency of transfection kinetics for a, b CBP siRNA in MCF7 and T47D cells. **Fig. S4.** Uncropped blots for a CBP and b GCN5 proteins in normal and cancer breast cells. **Fig. S5.** Uncropped blots for HER2 and CBP proteins in a SkBr3 and b BT-474 cells transfected with HER2 siRNA for 24-96 hours. **Fig. S6.** Uncropped blots for CBP protein in a SkBr3 and b BT-474 cells treated with Trastuzumab for 24-96 hours. **Fig. S7.** Uncropped blots for ER and CBP proteins in a MCF7 and b T47D cells transfected with ER siRNA for 24-96 hours. **Fig. S8.** Uncropped blots for ER and CBP proteins in a BT-474 cells transfected with ER siRNA for 24-96 hours. Uncropped blots for CBP protein in b MCF7, c T47D and d BT-474 cells treated with Tamoxifen for 24-96 hours. **Fig. S9.** Uncropped blots for ER, HER2 and CBP proteins in a BT-474 cells transfected with ER and HER2 siRNAs for 24-96 hours. Uncropped blots for CBP protein in b BT-474 cells treated with Tamoxifen and Trastuzumab combination for 24-96 hours. **Fig. S10.** Uncropped blots for ER and CBP proteins in a MCF7 and b T47D cells transfected with CBP siRNA for 24-96 hours. **Fig. S11.** Kaplan-Meier survival curves of disease-free survival for CBP expression in a Luminal A, b Luminal B HER2 negative, c Luminal B HER2 positive, d HER2-positive and e Triple negative breast cancer patients. **Fig. S12.** Kaplan-Meier survival curves of disease-free survival for GCN5 expression in a Luminal A, b Luminal B HER2 negative, c Luminal B HER2 positive, d HER2-positive and e Triple negative breast cancer patients.

## Data Availability

All data generated or analyzed during this study are included in this published article (and its Additional file 1).
