# Supplementary material for: The impact of CBP expression in estrogen receptor-positive breast cancer
Source: Clin Epigenetics. 2021 Apr 7;13:72. doi: 10.1186/s13148-021-01060-2 (PMC8028106; doi:10.1186/s13148-021-01060-2)
Supplement: Supplementary file 1 — Additional file 1. Supplementary Tables: Table S1. Molecular subtypes of breast cancer cell lines. Table S2. Histopathological features of tissue microarray samples. Table S3. Clinical-pathological parameters and CBP & GCN5 expression in DCIS and breast carcinoma cases. Supplementary Figures: Fig. S1. Baseline expression level of ER and HER2 in a panel of normal cancer breast cells. Fig. S2. Efficiency of transfection kinetics for a, b HER2 siRNA, c-e ER siRNA and f, g both ER and HER2 siRNAs. Fig. S3. Efficiency of transfection kinetics for a, b CBP siRNA in MCF7 and T47D cells. Fig. S4. Uncropped blots for a CBP and b GCN5 proteins in normal and cancer breast cells. Fig. S5. Uncropped blots for HER2 and CBP proteins in a SkBr3 and b BT-474 cells transfected with HER2 siRNA for 24-96 hours. Fig. S6. Uncropped blots for CBP protein in a SkBr3 and b BT-474 cells treated with Trastuzumab for 24-96 hours. Fig. S7. Uncropped blots for ER and CBP proteins in a MCF7 and b T47D cells transfected with ER siRNA for 24-96 hours. Fig. S8. Uncropped blots for ER and CBP proteins in a BT-474 cells transfected with ER siRNA for 24-96 hours. Uncropped blots for CBP protein in b MCF7, c T47D and d BT-474 cells treated with Tamoxifen for 24-96 hours. Fig. S9. Uncropped blots for ER, HER2 and CBP proteins in a BT-474 cells transfected with ER and HER2 siRNAs for 24-96 hours. Uncropped blots for CBP protein in b BT-474 cells treated with Tamoxifen and Trastuzumab combination for 24-96 hours. Fig. S10. Uncropped blots for ER and CBP proteins in a MCF7 and b T47D cells transfected with CBP siRNA for 24-96 hours. Fig. S11. Kaplan-Meier survival curves of disease-free survival for CBP expression in a Luminal A, b Luminal B HER2 negative, c Luminal B HER2 positive, d HER2-positive and e Triple negative breast cancer patients. Fig. S12. Kaplan-Meier survival curves of disease-free survival for GCN5 expression in a Luminal A, b Luminal B HER2 negative, c Luminal B HER2 positive, d HER2-posit [file 13148_2021_1060_MOESM1_ESM.pdf]

Table S1. Molecular subtypes of breast cancer cell lines

Cell line name	Molecular subtype
**HMEpC**	Normal
**HME1**	Normal
**MCF7**	Luminal A
**T47D**	Luminal A
**BT-474**	Luminal B HER2+
**SkBr3**	HER2-overexpressed (ER-, PR-)
**BT549**	Triple negative
**MDA-MB-231**	Triple negative
**MDA-MB-468**	Triple negative
**BT-20**	Triple negative
**Hs578T**	Triple negative

ER, Estrogen receptor; PR, Progesterone receptor; HER2, Human epidermal growth factor receptor 2

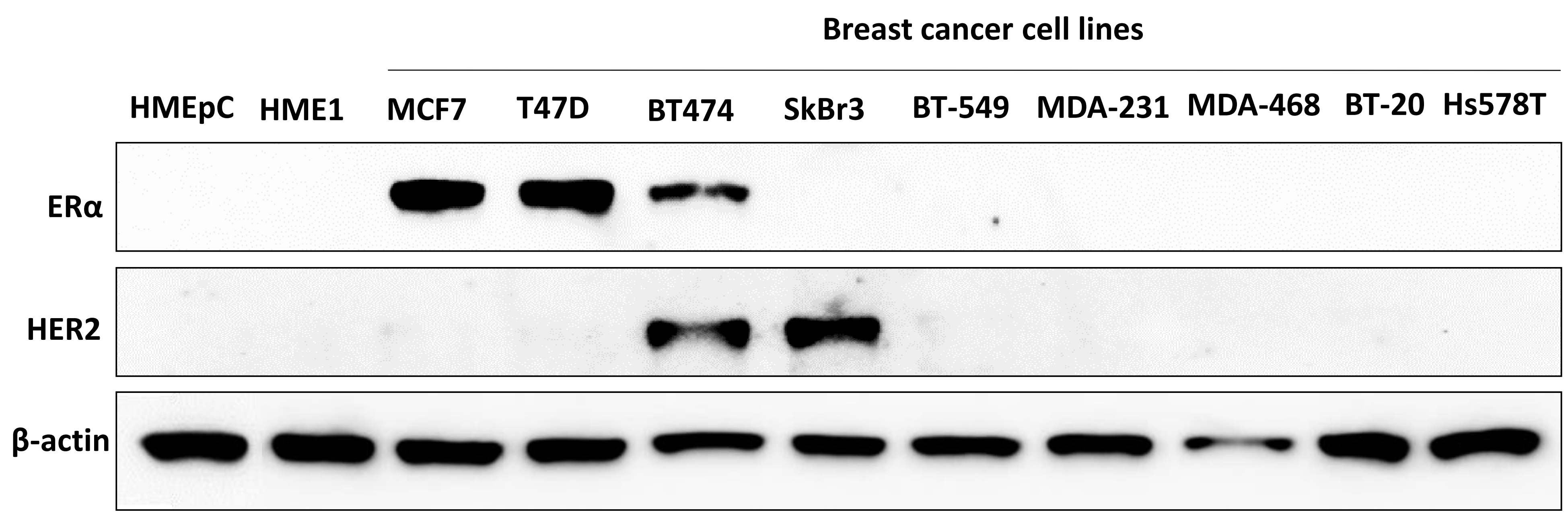

Fig. S1 Baseline expression level of ER and HER2 in a panel of normal cancer breast cells.

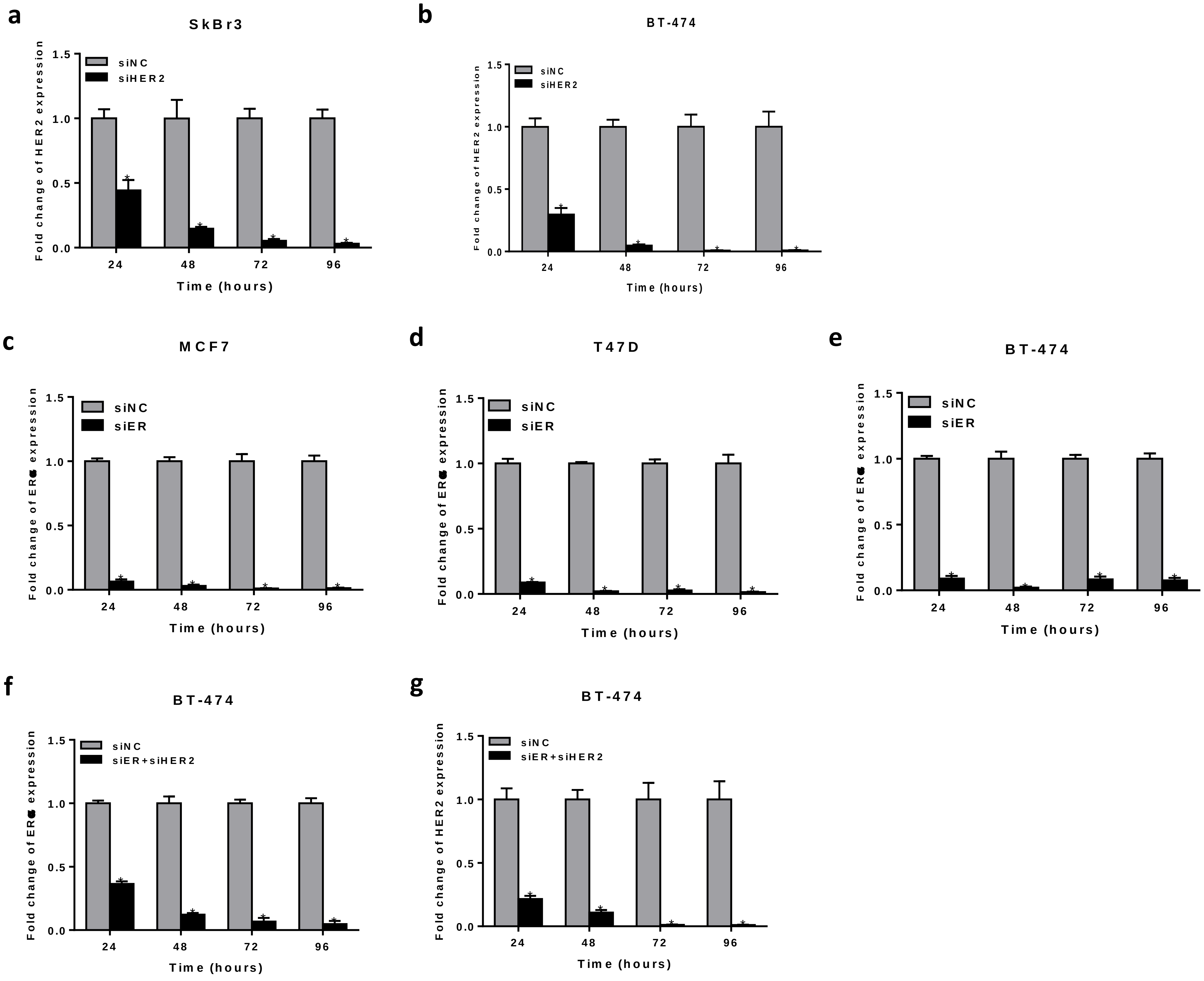

Fig. S2 Efficiency of transfection kinetics for **a, b** HER2 siRNA, **c-e** ER siRNA and **f, g** both ER and HER2 siRNAs

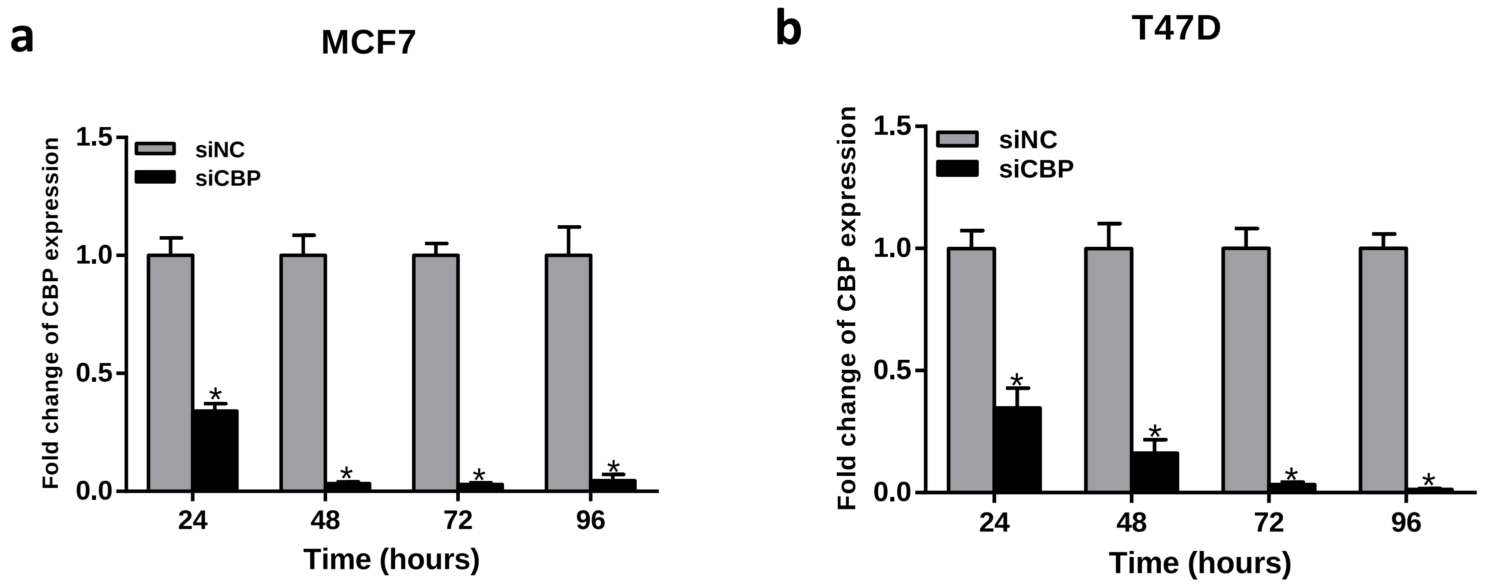

Fig. S3 Efficiency of transfection kinetics for **a, b** CBP siRNA in MCF7 and T47D cells.

**Table S2.** Histopathological features of tissue microarray samples	
**Histological subtypes**	**TMA samples**
**Normal tissue**	101
**Benign neoplasia**	45
**Ductal carcinoma in situ**	37
**Invasive carcinoma**	222
**Total**	405
TMA, Tissue microarray	

Table S3. Clinical-pathological parameters and CBP & GCN5 expression in DCIS and breast carcinoma cases

Variables	n	No. of Missing data (%)	CBP expression			n	No. of Missing Data (%)	GCN5 expression		
			Negative (%)[a]	Positive (%)[b]	*P*-Value[c]			Negative (%)[a]	Positive (%)[b]	*P*-Value[c]
**Age at diagnosis (years)** [d]										
≤40	14		2 (14.3)	12 (85.7)	0.750	15		2 (13.3)	13 (86.7)	0.951
41-70	125		18 (14.4)	107 (85.6)		125		18 (14.4)	107 (85.6)	
>70	69		7 (10.1)	62 (89.9)		70		11 (15.7)	59 (84.3)	
**Total**	**208**	1 (0.5)				**210**	1 (0.5)			
**Tumor Stage**										
pTis	6		1 (16.7)	5 (83.3)	0.117	6		1 (16.7)	5 (83.3)	0.440
pT1	49		9 (18.4)	40 (81.6)		50		9 (18)	41 (82)	
pT2	77		7 (9.1)	70 (90.9)		77		7 (9.1)	70 (90.1)	
pT3	8		3 (37.5)	5 (62.5)		8		2 (25)	6 (75)	
pT4	40		5 (12.5)	35 (87.5)		40		4 (10)	36 (90)	
**Total**	**180**	29 (13.9)				**181**	30 (14.2)			
**Histological grade**										
G1	8		0 (0)	8 (100)	0.413	7		0 (0)	7 (100)	0.710
G2	144		19 (13.2)	125 (86.8)		146		21 (14.4)	125 (85.6)	
G3	35		6 (17.1)	29 (82.9)		36		5 (13.9)	31 (86.1)	
**Total**	**187**	22 (10.5)				**189**	22 (10.4)			
**Lymph node metastasis**										
Negative	82		15 (18.3)	67 (81.7)	0.088	80		12 (15)	68 (85)	0.195
Positive	82		8 (9.8)	74 (90.2)		85		8 (9.4)	77 (90.6)	
**Total**	**164**	45 (21.5)				**165**	46 (21.8)			

[a] Negative: IRS 0 to 5, [b] Positive: IRS 6 to 9, [c] Chi-square test

a

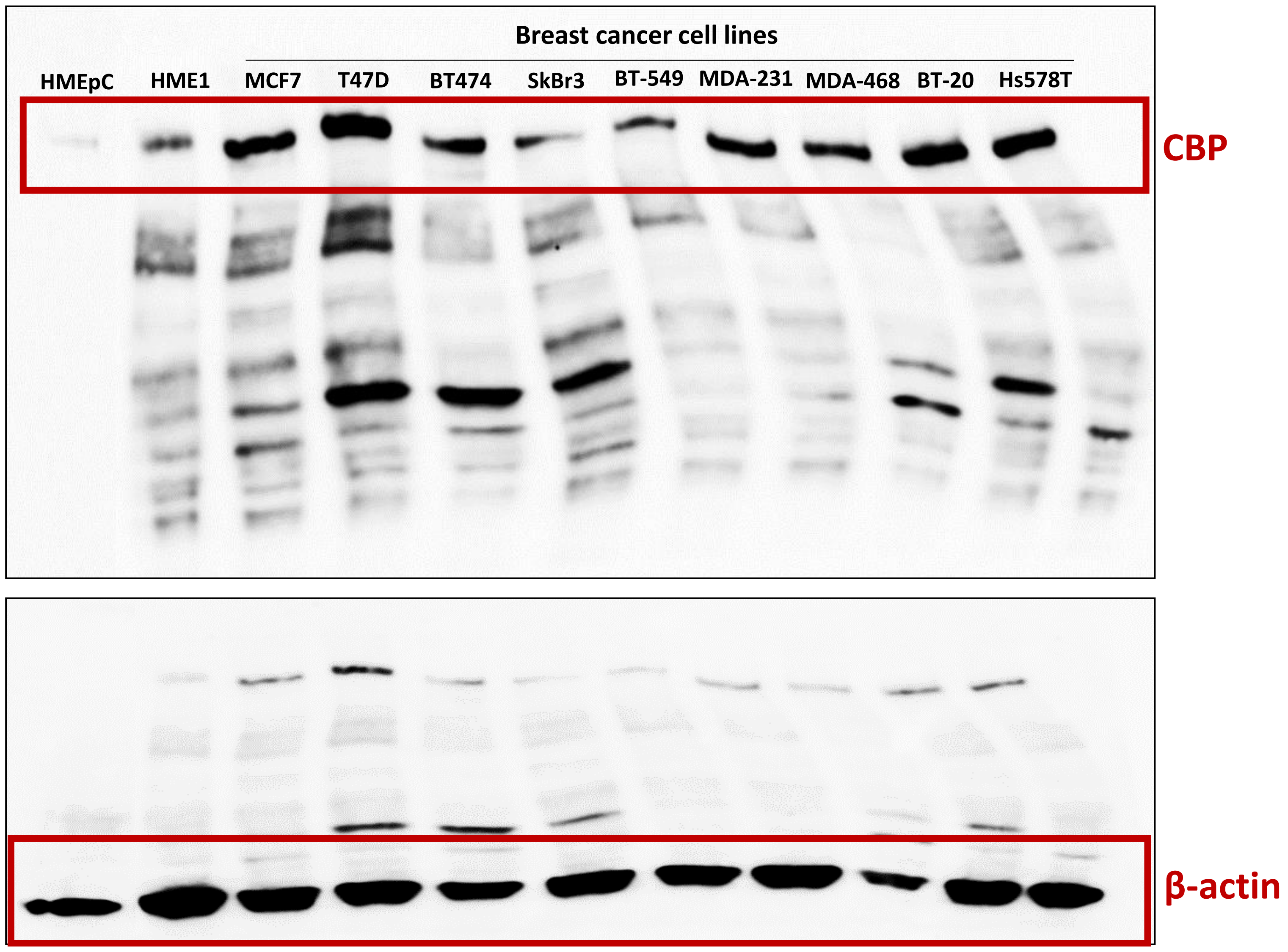

b

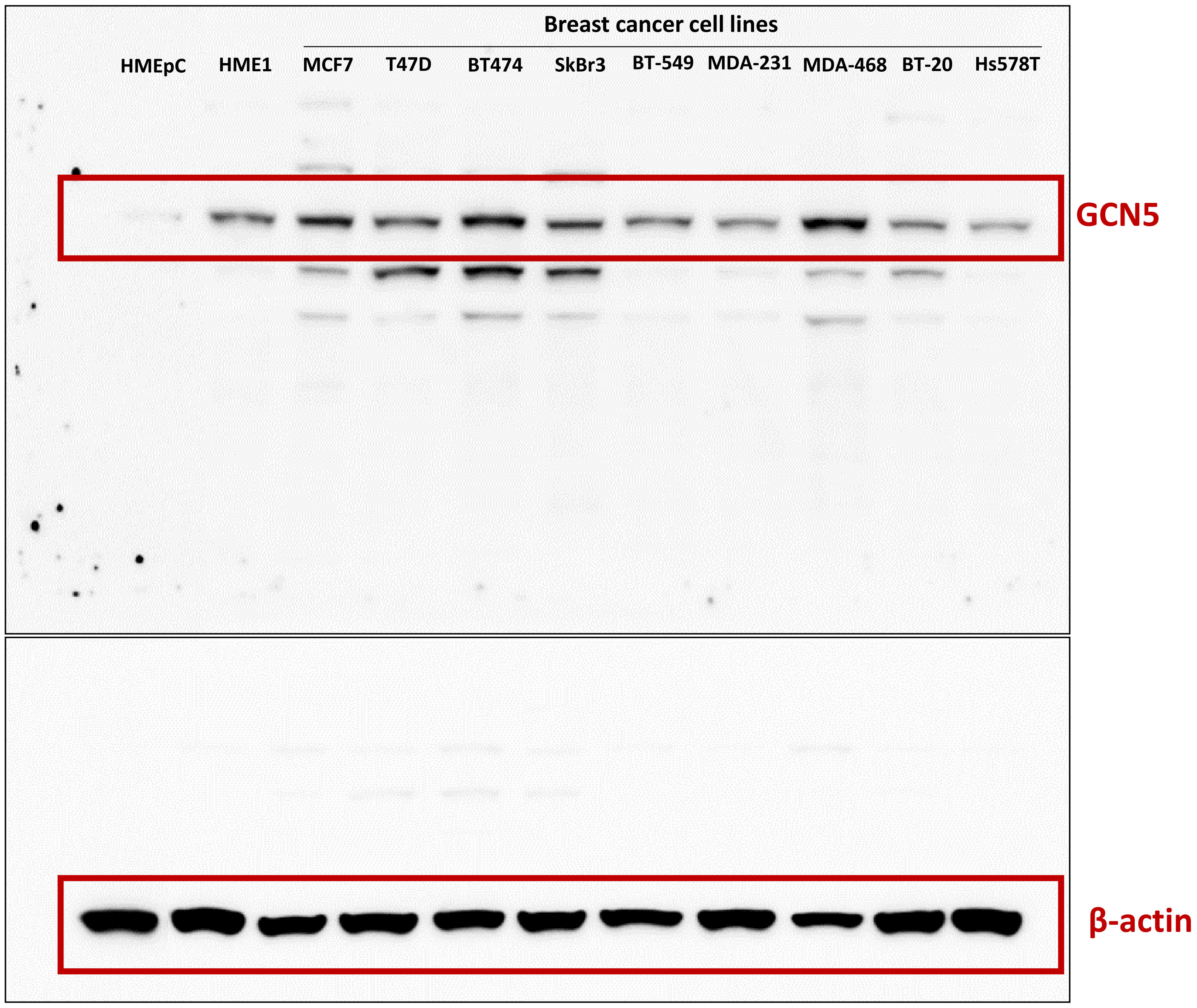

Fig. S4 Uncropped blots for **a** CBP and **b** GCN5 proteins in normal and cancer breast cells.

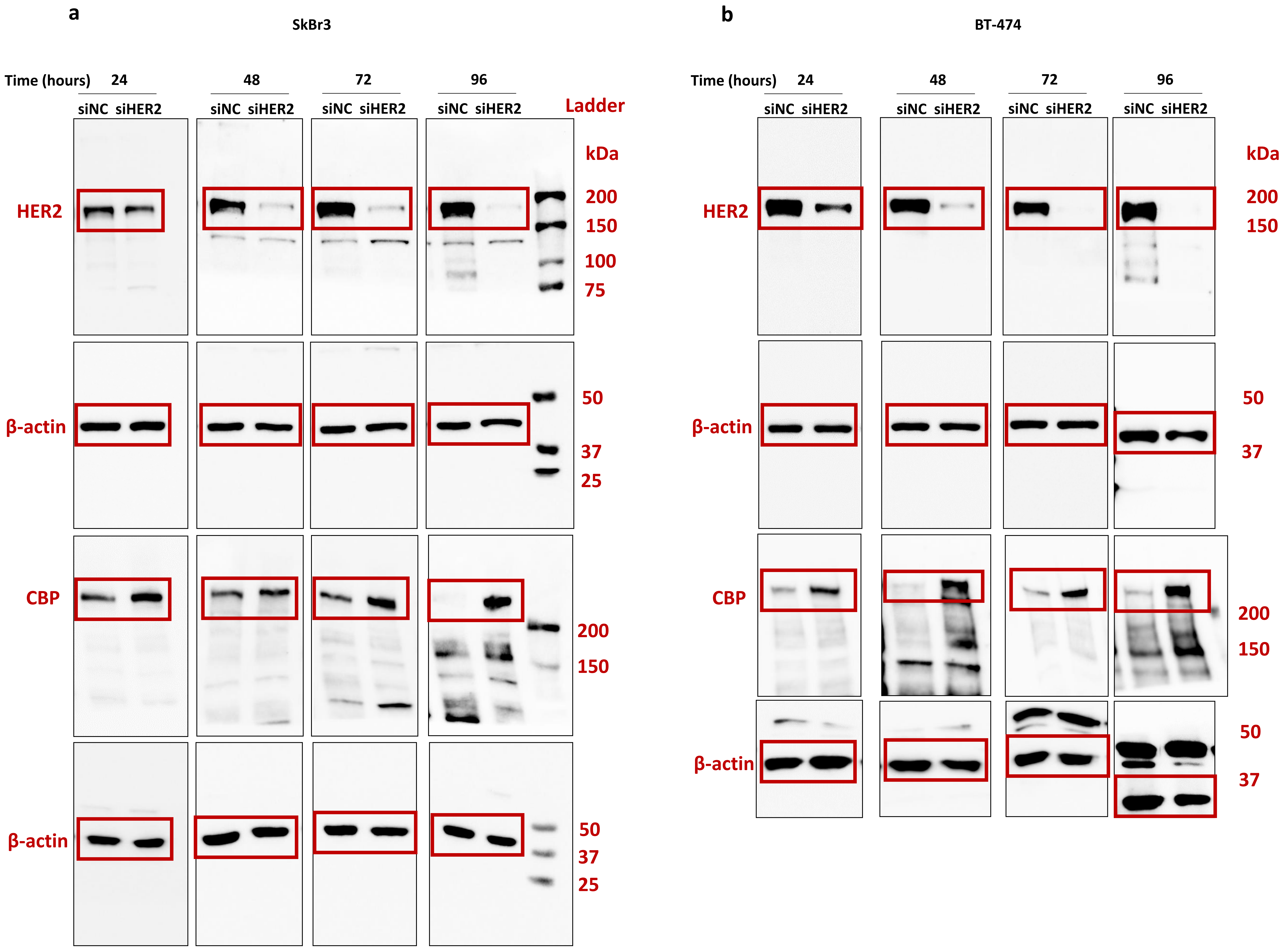

Fig. S5 Uncropped blots for HER2 and CBP proteins in **a** SkBr3 and **b** BT-474 cells transfected with HER2 siRNA for 24-96 hours.

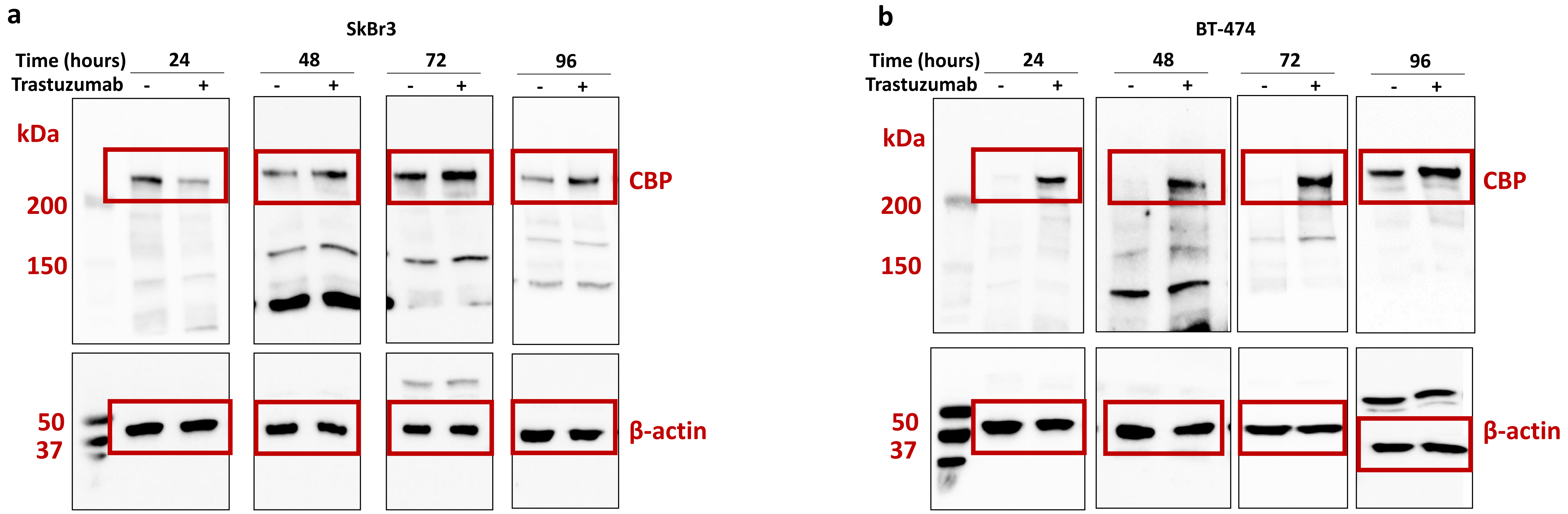

Fig. S6 Uncropped blots for CBP protein in **a** SkBr3 and **b** BT-474 cells treated with Trastuzumab for 24-96 hours.

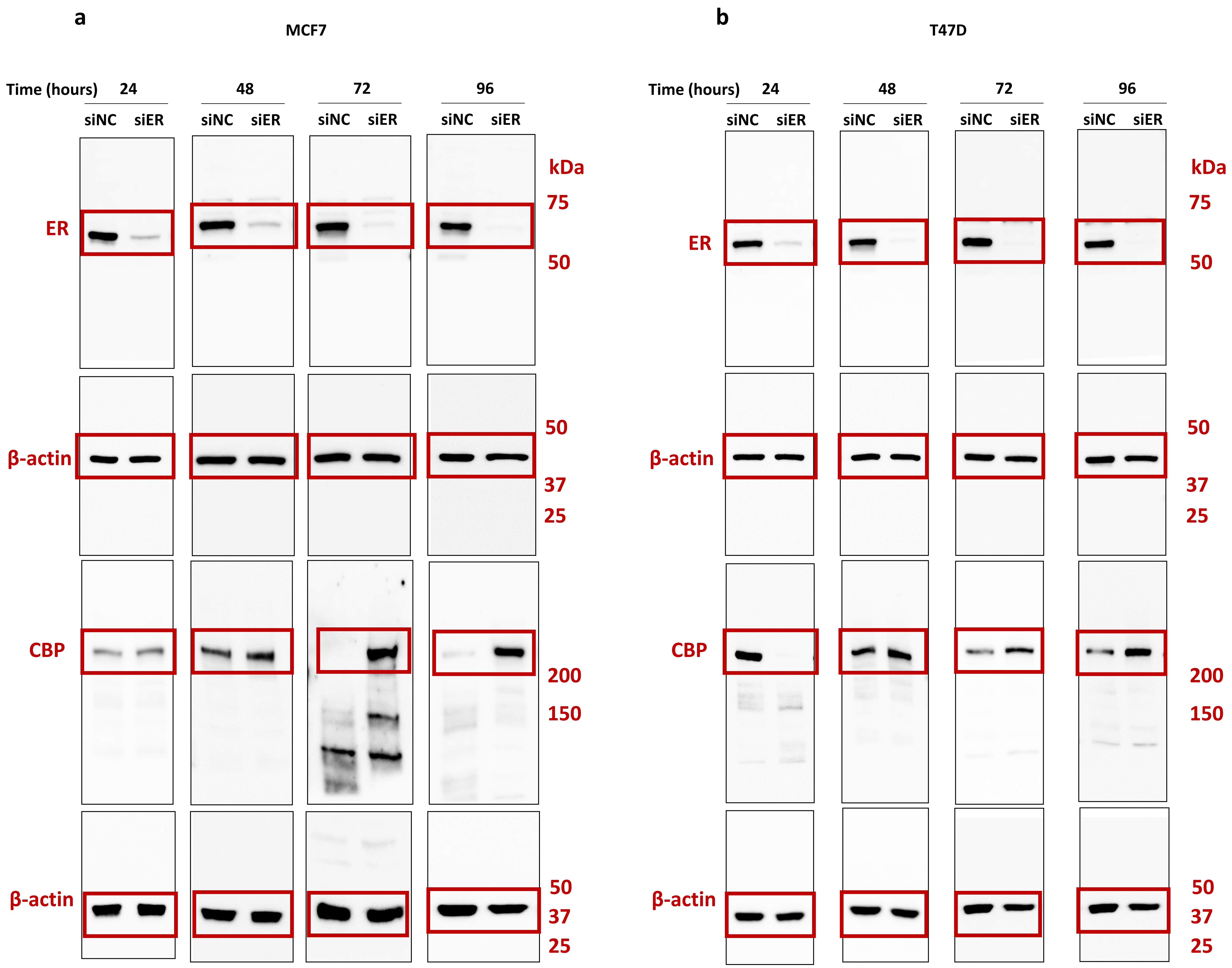

Fig. S7 Uncropped blots for ER and CBP proteins in **a** MCF7 and **b** T47D cells transfected with ER siRNA for 24-96 hours.

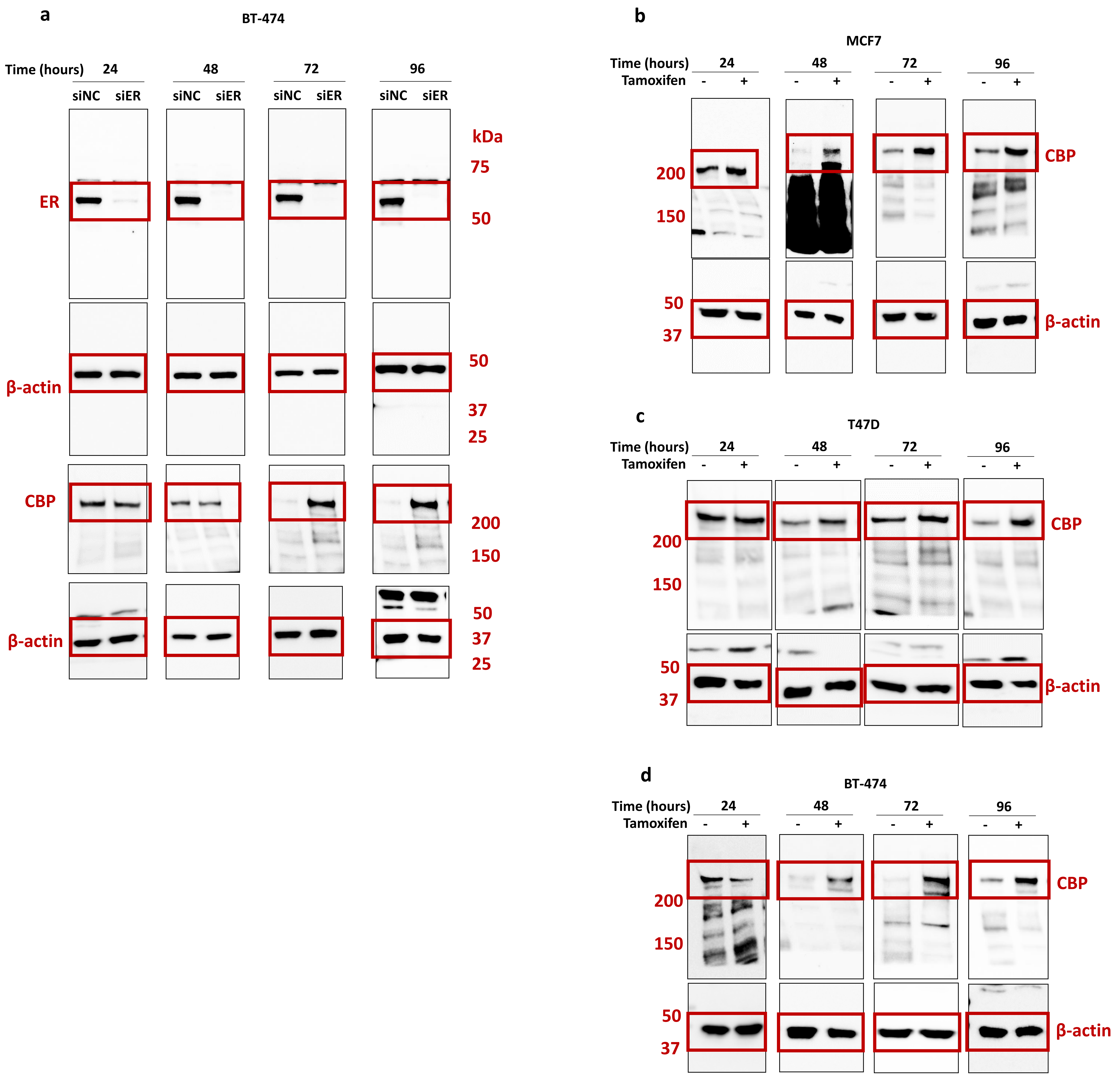

Fig. S8 Uncropped blots for ER and CBP proteins in **a** BT-474 cells transfected with ER siRNA for 24-96 hours. Uncropped blots for CBP protein in **b** MCF7, **c** T47D and **d** BT-474 cells treated with Tamoxifen for 24-96 hours.

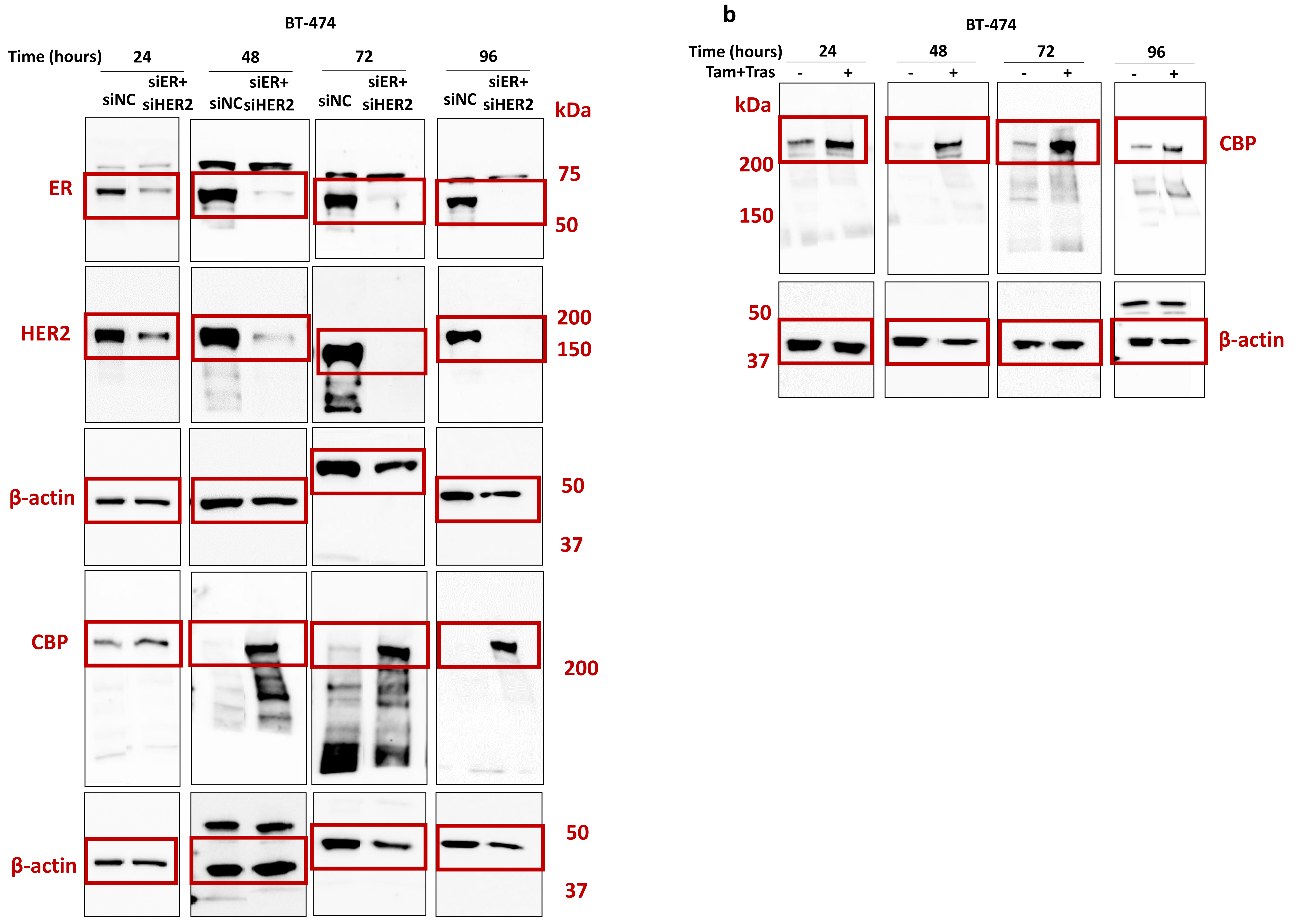

Fig. S9 Uncropped blots for ER, HER2 and CBP proteins in **a** BT-474 cells transfected with ER and HER2 siRNAs for 24-96 hours. Uncropped blots for CBP protein in **b** BT-474 cells treated with Tamoxifen and Trastuzumab combination for 24-96 hours.

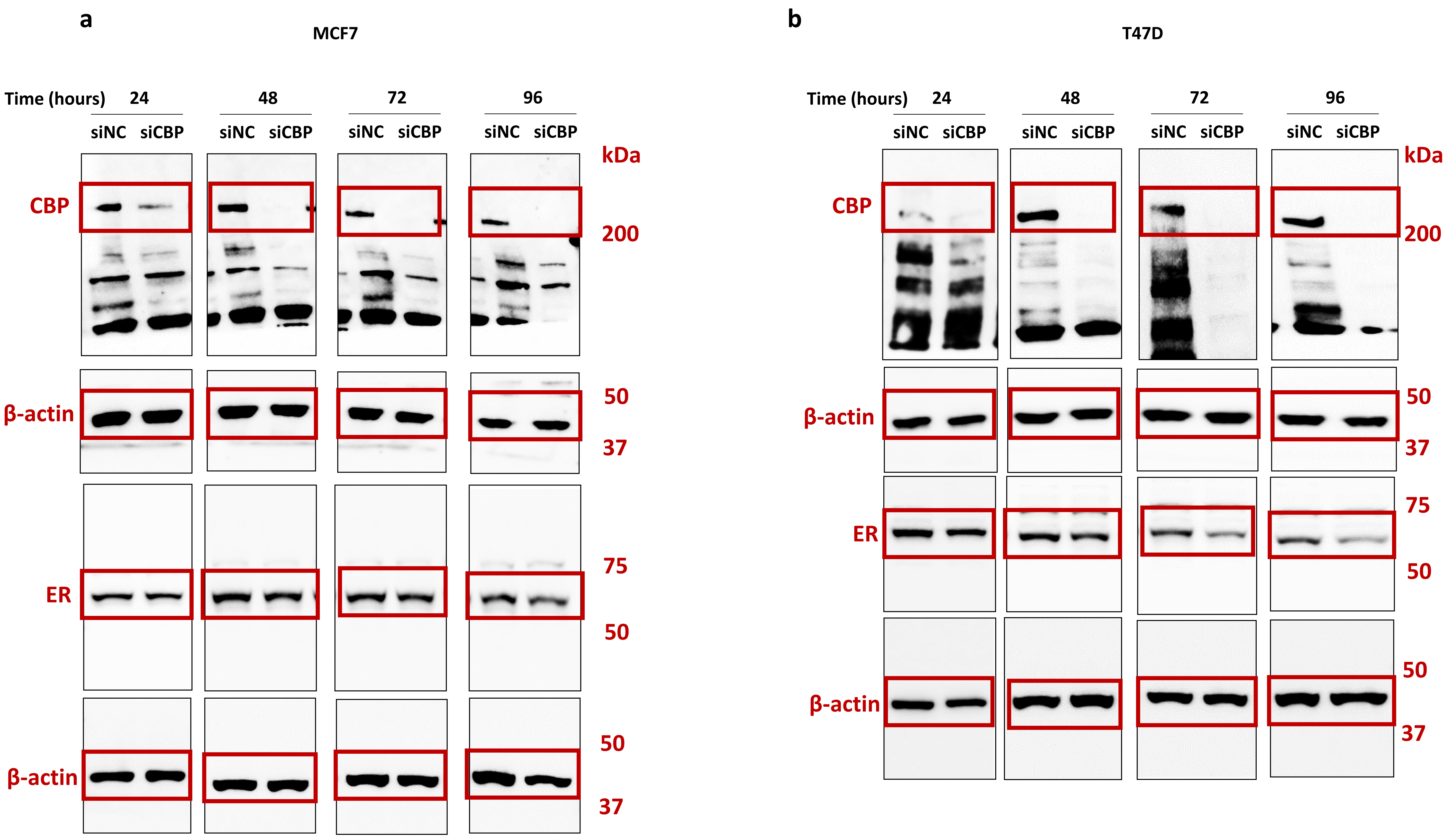

Fig. S10 Uncropped blots for ER and CBP proteins in **a** MCF7 and **b** T47D cells transfected with CBP siRNA for 24-96 hours.

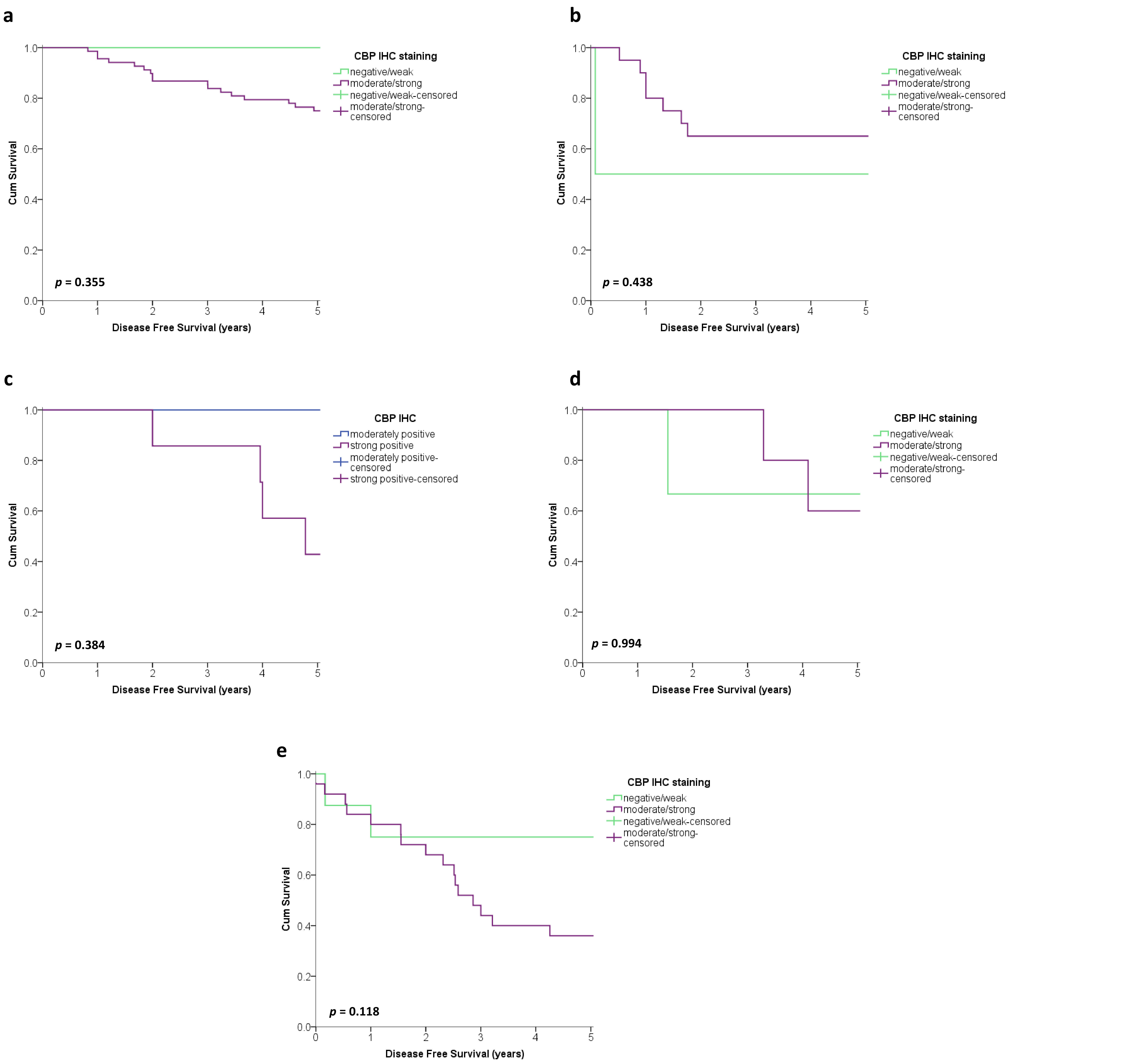

Fig. S11 Kaplan-Meier survival curves of disease-free survival for CBP expression in **a** Luminal A, **b** Luminal B HER2 negative, **c** Luminal B HER2 positive, **d** HER2-positive and **e** Triple negative breast cancer patients.

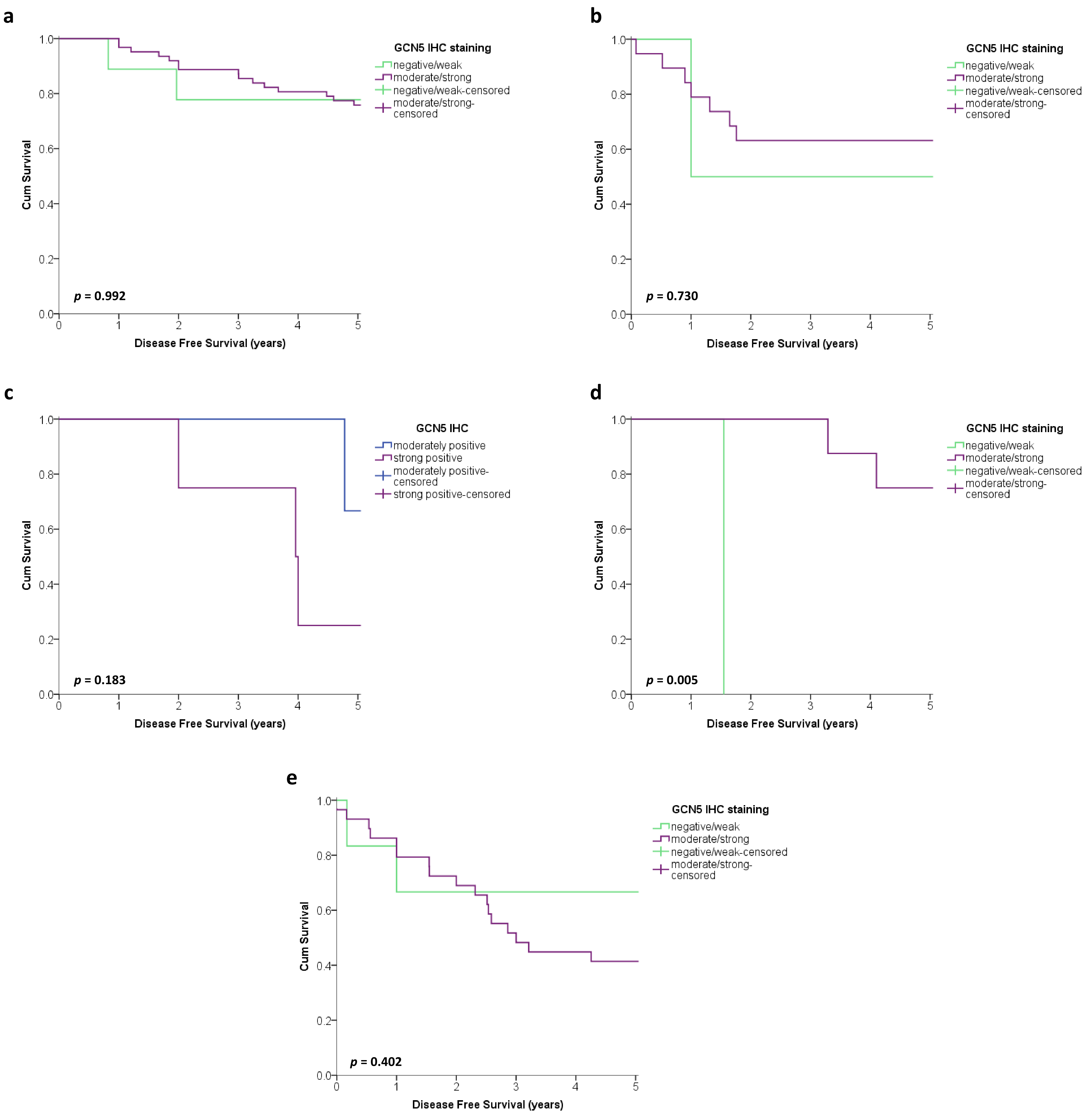

Fig. S12 Kaplan-Meier survival curves of disease-free survival for GCN5 expression in **a** Luminal A, **b** Luminal B HER2 negative, **c** Luminal B HER2 positive, **d** HER2-positive and **e** Triple negative breast cancer patients.
